# Stochastic Computing Convolutional Neural Network Architecture Reinvented for Highly Efficient Artificial Intelligence Workload on Field-Programmable Gate Array

**DOI:** 10.34133/research.0307

**Published:** 2024-03-04

**Authors:** Yang Yang Lee, Zaini Abdul Halim, Mohd Nadhir Ab Wahab, Tarik Adnan Almohamad

**Affiliations:** ^1^ School of Electrical and Electronic Engineering, Universiti Sains Malaysia, Nibong Tebal, 14300 Penang, Malaysia.; ^2^ School of Computer Sciences, Universiti Sains Malaysia, Gelugor, 11800 Penang, Malaysia.; ^3^ Electrical-Electronics Engineering Department, Faculty of Engineering, Karabuk University, 78050 Karabuk, Türkiye.

## Abstract

Stochastic computing (SC) has a substantial amount of study on application-specific integrated circuit (ASIC) design for artificial intelligence (AI) edge computing, especially the convolutional neural network (CNN) algorithm. However, SC has little to no optimization on field-programmable gate array (FPGA). Scaling up the ASIC logic without FPGA-oriented designs is inefficient, while aggregating thousands of bitstreams is still challenging in the conventional SC. This research has reinvented several FPGA-efficient 8-bit SC CNN computing architectures, i.e., SC multiplexer multiply-accumulate, multiply-accumulate function generator, and binary rectified linear unit, and successfully scaled and implemented a fully parallel CNN model on Kintex7 FPGA. The proposed SC hardware only compromises 0.14% accuracy compared to binary computing on the handwriting Modified National Institute of Standards and Technology classification task and achieved at least 99.72% energy saving per image feedforward and 31× more data throughput than modern hardware. Unique to SC, early decision termination pushed the performance baseline exponentially with minimum accuracy loss, making SC CNN extremely lucrative for AI edge computing but limited to classification tasks. The SC’s inherent noise heavily penalizes CNN regression performance, rendering SC unsuitable for regression tasks.

## Introduction

Stochastic computing (SC) is a computing methodology discovered in the 1960s when binary computing was expensive [1,2]. SC uses simple logic gates to perform the arithmetic operation by exploiting probability mathematics to compute in the probability domain. Nowadays, with exhaustive multiply-accumulate (MAC) operation in computing algorithms such as convolutional neural network (CNN) being deployed as part of artificial intelligence (AI) edge computing, binary computing architecture itself becomes the primary bottleneck due to limited memory bandwidth [3]. SC is regaining interest because it is suitable for AI computation, especially the CNN algorithm. SC has many studies in the field of application-specific integrated circuits (ASIC) due to its nature of the logic-level design. However, little attention has been allocated to readily accessible field-programmable gate array (FPGA). Putting AI workload on FPGA with SC architecture is even more difficult with limited modern software support.

SC bitstream’s significance or value is unknown until it is sampled over time. SC data is represented using probability distributions, and the operations performed on this data are based on probabilities rather than deterministic values. SC exploits probability mathematics to reduce the required logic gates to perform arithmetic. For example, a single AND gate could do multiplication on stochastic bitstreams in the SC domain because:

$$P\left(a\right)\times P\left(b\right)=P\left(a\cap b\right)=a\;\text{AND}\;b$$ (1)

The probability is the core of the SC. Thus, it is natural for SC to have noise and error, also known as approximate computing, because it could not be accurate. The only way to increase its accuracy is to increase its resolution or run length, i.e., progressive precision [1]. The output converges closer to the actual value with a longer bit stream. It could be helpful in applications where only the most significant digit matters, especially AI classification. Rising precision requires no hardware cost, but it increases the computing time exponentially for 2*^n^*, where *n* is the equivalent binary resolution, which is the main reason it dropped out in the face of binary computing. Nevertheless, approximate computing could be the next frontier of efficient computing [4], and SC has regained interest with the increasing demand for AI edge computing.

### The gateway to SC

**Fig. 1.**
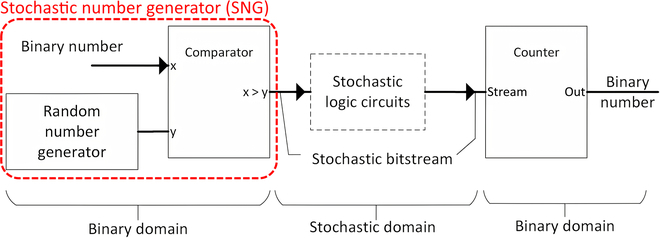
Process of SC.

**Fig. 2.**
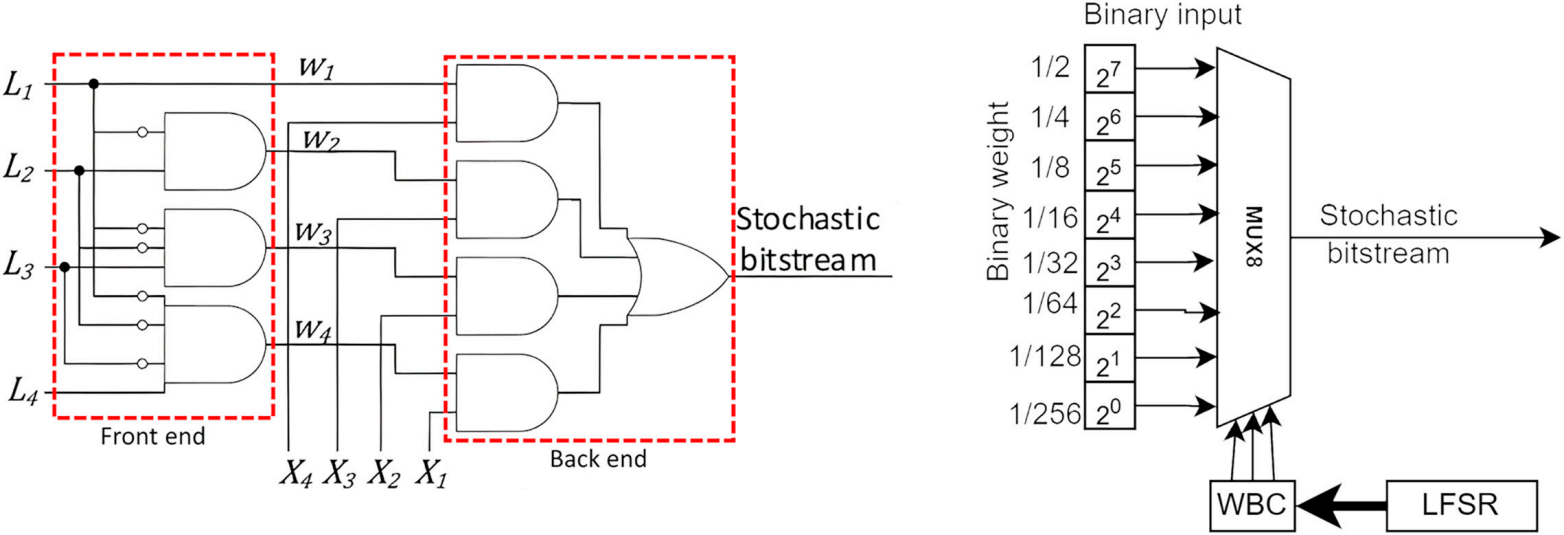
Conventional WBG (left) and MUX SNG driven by WBC (right).

To allow data to be processed in the SC domain, the binary data must be converted to stochastic bitstream via a stochastic number generator (SNG), as in Fig. 1, such that the number of 1’s bits represents the actual magnitude. Linear feedback shift register (LFSR) is widely adopted as a pseudo-random number generator for SNG, albeit other approaches exist, such as Sobol sequence generator [5–7] and deterministic sequence [8]. SNG itself could be a great bottleneck. Numerous works exist to lower SNG resource usage [9–15]. SNG is even being eliminated with wire spreading design [16,17], but it is unsuitable for FPGA. Interestingly, the digital multiplexer (MUX) could be repurposed for SNG [18–21], and [22] successfully upscaled the FPGA-friendly MUX to function as SNG via the weighted binary converter (WBC), reducing the SNG’s bottlenecking in FPGA as opposed to the former weighted binary generator (WBG) (in Fig. 2) in conventional SC.

### MAC operation in the SC domain

**Fig. 3.**
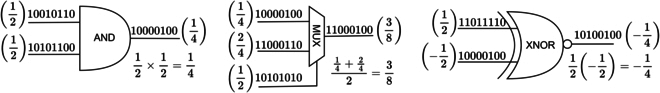
AND gate unipolar SC multiplier (left), MUX SC scaled adder (middle), and XNOR gate bipolar SC multiplier (right).

After the data is encoded into a probability stream, the data can be computed with simple logic gates, as shown in Fig. 3. AND gate (for unipolar SC, ∀ ∈ [0, 1]) and XNOR gate (for bipolar SC, ∀ ∈ [−1, 1], 0’s bit is encoded as −1) were adopted by almost all literature (except some literature that uses counters [19,23]) to accelerate multiplication operation. Logic gate arrangements could vary in some literature, such as binary as stochastic (integral SC) [24], extended stochastic logic (ESL) [25,26] or parallel streams for a single stochastic number [27]. However, the ASIC logic might not be equally area and power efficient on FPGA. For instance, implementing a single 2-input AND gate SC multiplier consumes half the look-up table (LUT) on Xilinx FPGA, which is not an efficient use of FPGA fabric since the LUT could fit any arbitrary n-input complex Boolean logic. Massively parallel gate arrays in conventional SC also could quickly congest the FPGA interconnect with thousands or millions of wires, limiting its scalability.

**Fig. 4.**
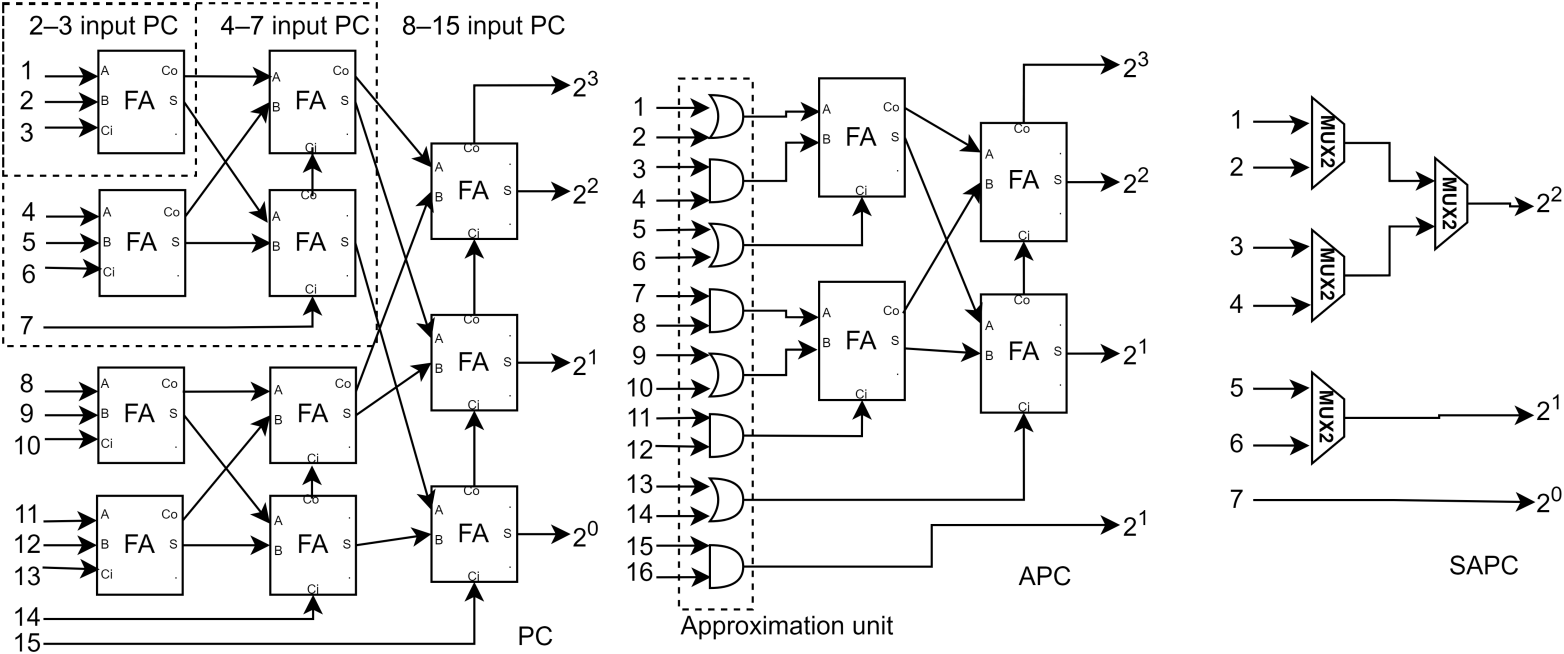
PC (left), APC (middle), and SAPC (right).

Multiplication is just part of the MAC operation. Aggregating thousands of bitstreams could still be challenging, taking 75% to 85% of total power consumption [28,29] in ASIC. Even though [30] proposed a more efficient approximate parallel counter (APC) (in Fig. 4) that uses less full adder (FA) than the parallel counter (PC) in ASICs and is being adopted by many studies, it might not save resources on FPGA because simple logic functions have low LUT utilization efficiency on FPGA. APC also suffered stochastic correlation complications [31], in which lowering correlation is favored for better accuracy in SC [1]. Meanwhile, [32] proposed MUX-tree as a stochastic APC (SAPC) but faces input size and run length trade-off with induced summation error. Efficiently aggregating thousands of bitstreams is still an open challenge.

### Activation functions in the SC domain

Nonlinear activation functions could be challenging in SC. A finite-state machine (FSM) is usually employed to implement hyperbolic tangent (Tanh) [1], rectified linear unit (ReLU) [32,33], and softmax [34] functions in SC. ReLU is easy with a comparator in the binary computing domain, but it is relatively inefficient in SC due to its probabilistic nature. It is unknown whether the bitstream is of positive value until being sampled. Li et al. [35] proposed pure SC ReLU hardware but worked in bipolar SC only, taking considerable resources by accumulating the bitstream and comparing it with a half-up counter. Sampling the magnitude and passing to the binary comparator induced huge latency [26,29]. Another approach is to convert the binary output of the APC back to the SC domain and exploit stochastic correlation to rule out positive bitstream. [36]. Regardless of the ReLU hardware designs of past literature, they suffered slow convergence speed and were resource intensive. Still, ReLU in SC yields better accuracy than other activation functions [37], so implementing the ReLU function more efficiently in the SC domain is of high interest. Sigmoid activation is possible in SC [38,39], but it mathematically compromises SC resolution.

### Problems of SC CNN on FPGA

Despite the design innovations, most research was biased toward ASIC design, and only a little is focused on FPGA. Even if the works are on FPGA, none of them revealed how exactly SC CNN on FPGA could be made scalable despite claiming that properties. The SC logic gate level design is yet to be optimized for FPGA. Most literature just forcibly transcoded ASIC logic without giving much attention to improving FPGA fabric’s utilization efficiency. Some SC functions remain challenging, especially bitstream accumulation and high-performing SC ReLU. Amid the increasing risk of chip shortages and the environmental impact of electronic waste, reconfigurable devices such as FPGA could be a good option for more sustainable development [40].

### Contributions

In this paper, a novel FPGA-scalable SC CNN architecture is reinvented and devised after revisiting the SC theory, contributing to the success of fully parallel CNN implementation on Xilinx Kintex7 XC7K325T FPGA. The novel FPGA-optimized 8-bit SC CNN architecture achieved state-of-art handwriting Modified National Institute of Standards and Technology (MNIST) classification performances in all aspects. The contributions are as follows:

• The proposed design increases the FPGA logic density by >2× and uses >6× fewer broadcast wires, improving SC scalability in FPGA.

• The proposed design eliminates bitstream aggregation inefficiency with a 2-staged accumulation process, saving up to 75% of resources than the conventional SC.

• The proposed design eradicates precision loss problems in the conventional SC by reinventing MUX as a scaling-free MAC operator.

• The proposed design accelerates SC ReLU function convergence at higher accuracy at zero hardware cost.

• The proposed design upscales the MUX SNG to 8-bit resolution by using the WBC [22], saving 62.5% registers in LFSR sharing and 83% of LUT than conventional WBG in implementing fixed weights.

## Results

A complete CNN feedforward took just 2.327 *μ*s, i.e., 256 clocks at 9.091-ns clock period, as shown in Table 1. Lower early decision termination (EDT) is possible since only 22 clocks are required to complete the binary data transfer via a 64-bit wide input port, exponentially pushing the energy efficiency line. SC CNN with EDT of 16 clocks and below are simulated with MATLAB for comparison study since the FPGA EDT is limited to a minimum of 23 clocks (additional 1 clock for global reset). The SC bit has virtually zero latency passing through all CNN layers when a huge feedforward amount of image batch is executed, achieving zero memory bottlenecking. Only the real power that contributes to the CNN computation is of interest. Hence the total power of the binary computing was deducted with its power draw in idle, while the power from FPGA is the reported on-chip power draw from postimplementation.

**Table 1.** Power, speed, and energy efficiency of single 28 × 28 × 1 image inference for handwriting MNIST classification task

Platform /device	Compute domain /variant	Time /image	Real power (W)	Energy /image	Note
MATLAB R2022a	Binary (CPU)	72.9 μs	79.4	5.79 mJ	Intel i7-11700F CPU, factory setting, batch size = 10,000
	Binary (GPU)	17.2 μ*s*	66.0	1.14 mJ	Nvidia RTX3090 GPU, power limit @28%, batch size = 10,000
	SC (simulate)	1.31 s	79.5	104 J	Multithreaded vector computing on all core
C++ Xilinx Vitis HLS	SC (emulated)	20.13 s	77.6	1,562 J	Single-threaded clock-wise SC emulation, disabled file I/Os
FPGA (Xilinx Kintex7 XC7K325T @ 110MHz)	SC	2.33 μ*s*	6.757	15.72 μJ	Postimplementation simulation, full SC
		1.16 μ*s*		7.87 μJ	Postimplementation simulation, EDT = 128
		0.58 μ*s*		3.93 μJ	Postimplementation simulation, EDT = 64
		0.29 μ*s*		1.97 μJ	Postimplementation simulation, EDT = 32

**Fig. 5.**
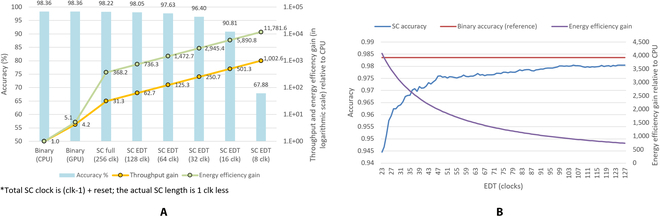
(A) Accuracy, speed, and energy efficiency versus computing domain for handwriting MNIST image classification CNN. Throughput and energy efficiency gain increase exponentially while having minimum impact on accuracy. (B) Accuracy and energy efficiency gain of the handwriting MNIST SC CNN in the EDT of 23 to 127 clocks.

**Fig. 6.**
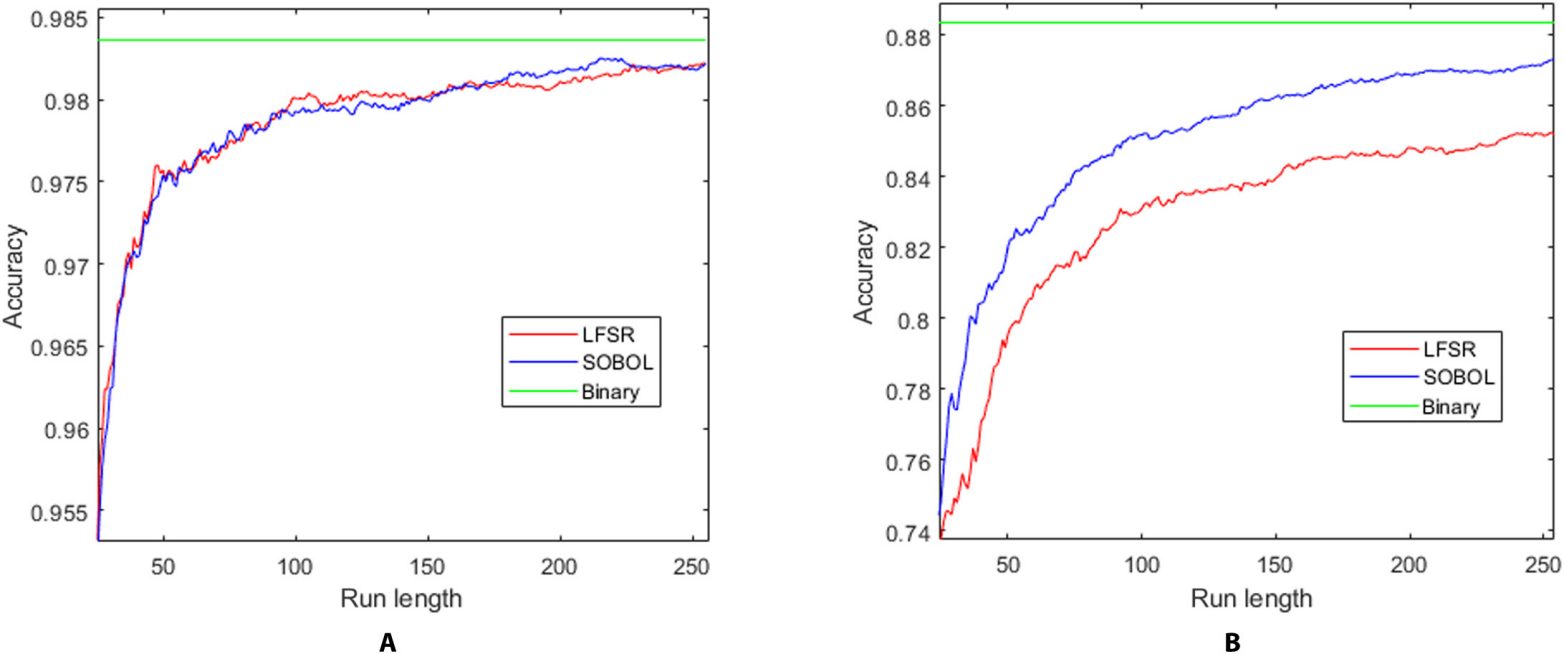
(A) SC CNN of LFSR and Sobol SNGs converged equally in handwriting MNIST, but the Sobol sequence has reached 0.11% lowest accuracy degradation at 215 clock cycle. (B) Sobol sequence pulls ahead of LFSR with faster convergence and higher accuracy in the fashion MNIST SC CNN simulation.

The astronomical leap in speed and energy efficiency is substantial. Figure 5A shows that the FPGA is already 5.79 mJ/15.72 *μ*J = 368 times more energy efficient than central processing unit (CPU) by default with full SC CNN run length. In other words, the SC CNN uses at least 100(1 − 1/368) = 99.72% less energy than binary computing CNN per image feedforward while degrading only 0.14% classification accuracy (as in Fig. 6A of the confusion matrix). In perspective, if binary computing uses 1 W to process images via CNN, the SC CNN implemented on FPGA would use only 2.7 mW to process the same number of images while only losing 0.14% accuracy, in addition to 72.9 *μ*s/2.33 *μ*s = 31 times higher data processing speed. Assuming SC EDT of 32 clocks at 1.96% accuracy degradation is acceptable, a 2,945× efficiency gain over the binary domain would translate to 99.97% of energy saving (just 340 *μ*W out of 1 W of binary computing, or 340 mW out of 1 kW in the server environment). Thus, the SC EDT capability is highly lucrative for Internet of Things and server applications by exploiting SC’s progressive precision mechanism.

**Fig. 7.**
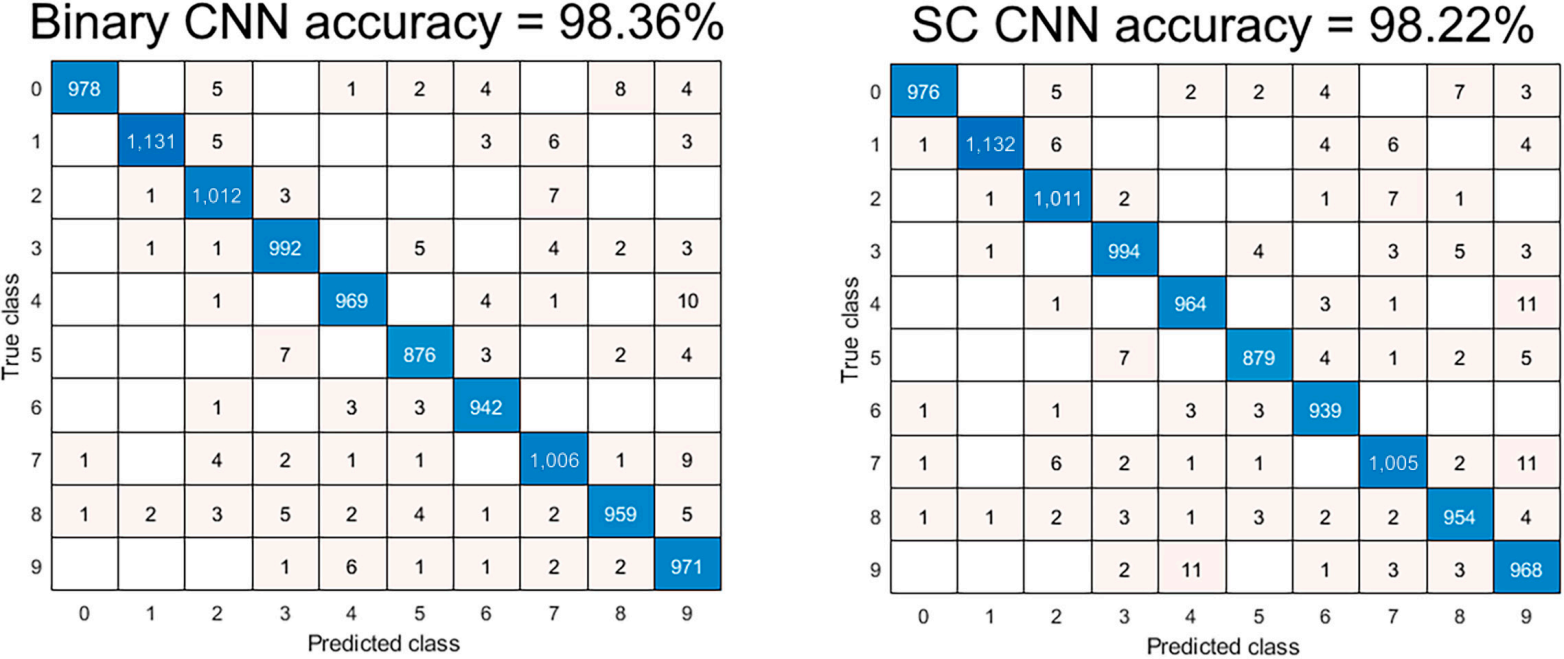
Confusion matrix of handwriting MNIST CNN classification task, binary versus SC domains.

Misclassification error increased when EDT was enabled but showed no significant difference, as shown in Fig. 5B. Even if the EDT is pushed to the absolute hardware limit, i.e., 23 clocks, it only loses 3.91% accuracy, as opposed to 5% loss at 71 clocks of unary general matrix multiplication [41] on the same handwriting MNIST dataset. Although not being implemented, the Sobol sequence via the least significant zero (LSZ) of binary up counting [5] could be fed to the input pixel MUX SNG, achieving a minimum 0.11% accuracy degradation at 215 clocks in the simulation, as shown in Fig. 7. The novel SC binary ReLU (BReLU) architecture takes multiple bitstreams to accelerate convergence, greatly improving the convergence time of LFSR-based SC. The multiply-accumulate function generator (MACFG) programmed the SC multiplexer multiply-accumulate (MMAC) operation in pseudo-random order and evenly distributed the output bitstream information across the SC run length. Hence, the convergence outcomes are predictable even in extremely short EDT. The novel SC BReLU works only if the bitstream is random (unsuitable for the deterministic SC approach) because it only measures the biases of random distribution. The SC BReLU state size is set to 5-bit because of diminishing 0.04% improvement at most in 8-bit state size in the simulation.

Comparing with other works of handwriting MNIST classification on FPGA, as listed in Table 2, shows that the SC CNN hardware architecture is over 4.5× more energy efficient and 4× area efficient than [36], delivering 46.1% higher inference speed and 7× lower accuracy degradation. To closely match the 1% accuracy degradation of [36], the SC MMAC could have the EDT target of 64 clocks at 0.73% accuracy degradation, achieving 18× and 17× energy and area efficiency gain, respectively. Comparing with the SC ESL architecture of [26] shows that the SC MMAC at a similar run length is less energy and area efficient because [26] utilized folding technique, i.e., reusing FPGA hardware for different layers (in their case is fully connected neuron [FCN] layer), while their FPGA platform is newer. However, the SC ESL suffered a great accuracy loss of 2.24% and a much higher latency. The SC MMAC could easily compensate for the energy and area deficiencies by having EDT = 128 while still achieving 7× lower accuracy degradation than SC ESL. SC ESL at 1,024-bit run length achieved only a 1.48% accuracy loss at best but severely compromised the energy and area efficiency. Comparing with the integral SC [24] shows that the SC MMAC achieved way lower accuracy degradation with relatively simpler SC logic.

**Table 2.** Handwriting MNIST classification on FPGA compared to different works

Reference	FPGA1 [24]	FPGA2 [64]	FPGA3 [65]	FPGA4 [66]	FPGA5 [67]	FPGA6 [44]	FPGA7 [36]	FPGA8 [26]	This research
Year	2017	2018	2018	2020	2021	2021	2021	2023	2024
Hardware architecture	Integral SC	Binary (8-bit)	Binary	Neuro- morphic	Binary (8-bit)	Binary (3-bit)	SC (parallel)	SC ESL (sequential)	SC MMAC (parallel
AI model	FCN	CNN	CNN (LeNet-5)	CNN	CNN (LeNet-5)	FCN	CNN (LeNet-5)	FCN	CNN
FPGA platform	Virtex7 XC7V2000T	Virtex7 485T	Zynq ZC706	Artix7 100T	Zynq XCZU9EG-2ffvb1156	Zynq Z-7020	Arria 10 GX1150	Virtex7 XC7V2000T	Kintex7 XC7K325T
Frequency (MHz)	NS	NS	166	160	100	NS	150	100	110
Run length (bit)	1,024	NA	NA	NA	NA	NA	256	1,024 /256	256 /128 /64 /32
Latency (μs)	6.503	960	1,607	157.8	5,127	NS	3.4	61.38 /15.3	2.327 /1.164 /0.582 /0.291
Kilo-inferences/ second (KIPS)	NS	1.041	0.622	6.337	NS	372.877	294.1	16.291 /65.359	429.688 /859.366 /1,718.8 /3,436.4
Performance (KIPS/MHz)	NS	NS	0.00375	0.0396	NS	NS	1.960	0.163 /0.654	3.906 /7.812 /15.625 /31.24
Power (W)	NS	0.47	10.98	0.59	1.673	NS	21	0.798 /0.74	6.757
Energy efficiency (KI/J)	NS	2.21	0.056	10.74	NS	NS	14	20.41 /88.32	63.6 /127.2 /254.4 /508.6
Logic used (LUT)	437.461k	7204	39.837k	49k	57.657k	±1,2236 ^a^	343.4k ^b^	19.276k /14.964k	153.151k
DSPs used	NS	574	59	NS	123	NS	0	NS	0
Memory blocks used	NS	343.5	97	NS	102	NS	0	NS	0
Area efficiency (Inferences /MHz /(LUT6 or ALMa)	NS	NS	8 × 10^−5^	8 × 10^−4^	NS	NS	0.006 ^b^	0.008 /0.044	0.026 /0.051 /0.102 /0.204
Accuracy degradation / loss (%)	2.33	1.01	NS	0.2	0.38	3.5	1	1.48 /2.24	0.14 /0.31 /0.73 /1.96

*^a^*Recalculated from given percentages.

*^b^* Intel FPGA fabric is of LUT4 architecture, not an exact apple-to-apple comparison to Xilinx LUT6.

NS, not specified; NA, not applicable.

Comparing with the open-source FINN deep learning dataflow compiler from the official Xilinx Research Labs [42–44] (labeled as FPGA6 in Table 2) shows that the SC CNN is superior to the binary computing quantized CNN, achieving way less accuracy degradation with much higher effective resolution at higher throughput. To closely match the Xilinx FINN’s 3.5% accuracy degradation of [44], the SC CNN could have stopped the computation at an EDT of 25 clocks at 3.2% accuracy degradation, reaching 110 MHz/25 = 4.4 million images per second (>11× of the FINN’s throughput). It might not be a fair comparison since the FINN is at 3-bit resolution while the SC CNN is at 8-bit.

Simulation result on the more challenging fashion-MNIST dataset shows that the SC CNN has attained 85.78% accuracy compared to the binary counterpart of 88.35%, i.e., 2.57% accuracy degradation on LFSR SNG. Simulated Sobol SNG on the input pixel further improved the accuracy degradation to 1.05%, as shown in Fig. 6B. Apart from the limited 8-bit SC resolution, the current simple CNN model also leads to poorer classification performance. Only the handwriting MNIST SC CNN made its way to the FPGA implementation. Nevertheless, component-wise study shows that the SC MMAC has superior resource efficiency than the equivalent binary MAC. The binary input, weight, and bias are 9-bit signed integers, and the output is of 16-bit fixed point (8-bit fraction, 7-bit integer, 1-bit sign), while the SC intermediate output is of 2-bit binary with half-adder PC to sum up the bias input. Table 3 shows that the 32-input SC MAC component is 332× and 35× more resource-efficient than non-DSP and DSP versions of the binary MAC equivalent, respectively, on the same FPGA. Depending on the use case, the latency of the SC might not be critical with the EDT capability if the application favors the resource efficiency of the SC domain.

**Table 3.** Component-wise synthesis outcome of SC MMAC and the equivalent binary MAC hardware on the same Kintex7 FPGA

Number of	Binary MAC										Half adder + SC MMAC + MACFG				
	DSP					No DSP									
Input	2	4	8	16	32	2	4	8	16	32	2	4	8	16	32
Weight	2	4	8	16	32	2	4	8	16	32	2	4	8	16	32
Bias	1	1	1	1	1	1	1	1	1	1	1	1	1	1	1
Output	1	1	1	1	1	1	1	1	1	1	1	1	1	1	1
LUT	19	39	80	160	320	190	380	758	1,497	2,993	1	3	3	6	9
FF	0	0	24	36	60	26	63	106	54	98	0	0	0	0	0
DSP	2	4	8	16	32	0	0	0	0	0	0	0	0	0	0
Latency	1					1	2	2	1	1	255				

### The potential application of the SC CNN

The ultralow latency of the SC CNN could be important for mission-critical applications such as a first-level trigger for particle accelerator’s data acquisition systems, where it only has a few clock cycles of time to make a decision. In the case of the newer ResNet [45] CNN design, the CNN’s residue map could be bypassed with simple shift registers since the interlayer SC CNN feedforward only costs 2 to 3 clock cycles, potentially reducing the memory resources than binary computing. Since random number sources drive the entire circuit, it is suitable for deep-space or high-radioactivity environments. Random bit flips in single-event upset caused by energetic particles could be catastrophic to binary computing but would not affect the SC circuit’s functionality. For instance, it could be deployed on robotic probes surveying the Fukushima nuclear reactor meltdown, and the probes could have survived the intense radioactivity. Its ultrahigh energy efficiency also fits deep-space probes or robots of extremely limited power budget, enabling wider AI adoption in the future space industry.

### An attempt to implement SC CNN for a more realistic AI workload

**Fig. 8.**
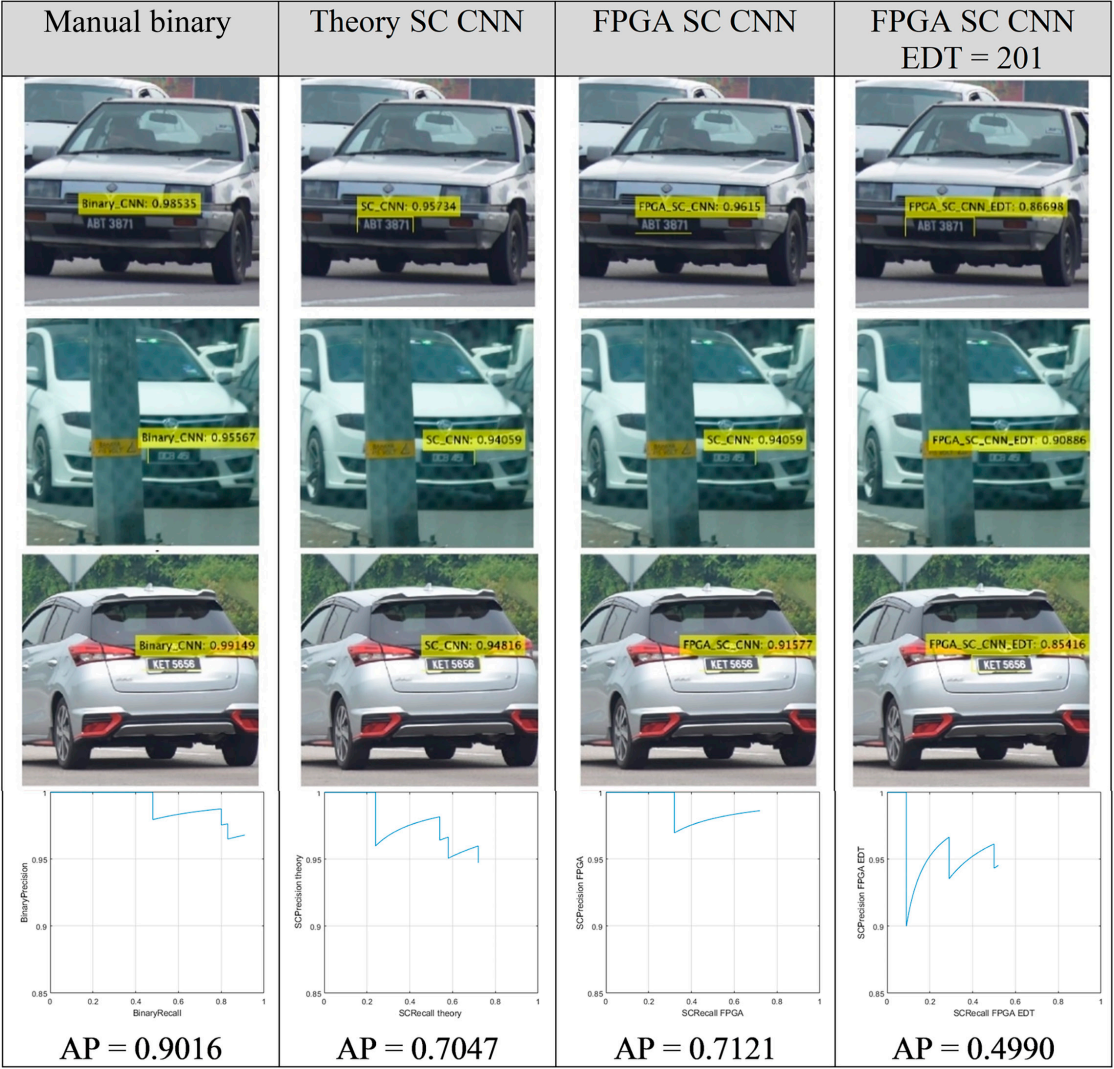
Visual inspection of the ALPR YOLOv3 detection and the AP curve of each method over 100 test images. SC CNN causes the AP scores to degrade by about 20%. Enabling EDT only worsens the effect.

Another side development has been attempted to integrate SC CNN to the front end of the YOLOv3 (You Only Look Once) algorithm [46] for automated license plate recognition (ALPR) in extending the existing work from [47]. SC is proven attractive in power efficiency and speed, which suit the harsh edge computing requirements for ALPR applications. This is the first attempt to apply SC in ALPR integration for CNN bounding box regression tasks. In APLR, the location of the license plate and its characters has to be recovered via image processing techniques, and YOLO is one of the AI algorithms available for this task. However, it was found that the SC’s inherent noise inhibited the average precision (AP) score performance. Although energy efficiency and speed are promising, i.e., 1,054× and 77× gains than CPU, respectively, the AP score is severely degraded by 20% due to lower recall rates. The sigmoid activation of the YOLOv3’s objectness function is a great filter for SC noise. Longer SC run length could improve the numeric precision and lowers the noise, but the FPGA’s hardware limitation and the underdeveloped software ecosystem for SC CNN hindered the SC CNN development capacity, limiting the CNN model size for the ALPR application. SC is favorable for CNN classification tasks, not for bounding box regression tasks (in Fig. 8), at least for now.

## Conclusion

In this research, a scalable and programmable SC CNN architecture has been introduced. It greatly improved the scalability of the existing SC CNN methodology on FPGA. Novel SC architectures are proposed, i.e., SC MMAC, MACFG, and SC BReLU, and are successfully implemented on FPGA. The result shows that the SC CNN is at least 368× more energy efficient and at least 31× more data throughput than binary computing systems at a low 0.14% classification error cost. Energy efficiency and speed improved exponentially with SC EDT at minimal classification accuracy loss. SC is exceptionally favorable for CNN classification tasks, enabling a new realm of fast and efficient AI edge computing applications. The SC CNN handwriting MNIST classification achieved state-of-art performances on FPGA hardware. Nevertheless, it is unsuitable for the CNN regression task due to its inherent noise property, which is hard to eliminate.

Although it is proven feasible to implement a fully parallel CNN model via the SC domain, resource-sharing methods such as layer folding should allow SC to scale further. Replacing the LFSR with the Sobol sequence generator could improve the accuracy. FPGA’s digital signal processors (DSPs) do support bitwise operation and could have been utilized to reduce LUT usage. More aggressive EDT could have been achieved with high-speed interfaces like Advanced eXtensible Interface (AXI) or Peripheral Component Interconnect Express (PCIe). The CNN’s normalization layer could improve accuracy, but it is not yet resource-efficient in the SC domain. SC CNN requires immense software–hardware codesign effort and software automation, especially with FPGA development, which would be the major focus of future research.

## Methods

Unlike deterministic binary computing, SC relies on probability. The SC theory could only be understood from the perspective of probability mathematics. In some cases, binary computing could complement the SC circuit, so mixed domain use cases exist. Review papers published by [48,49] are a good knowledge base for SC before diving deep into the research methodology. Firstly, the SNG was an evident design bottleneck for SC since FPGA has limited resources. Thus, [22] proposed WBC to port the good old LFSR to Xilinx’s FPGA-friendly MUX, reducing the FPGA LUT resource usage and SNG broadcasting bit width by at least 33% and 50%, respectively, compared to ASIC logic. Nonetheless, MAC operation and the activation function have yet to be optimized for FPGA, which leads to a question of whether the MUX could do more than just being an SNG or if there is a programmable way with universal SC CNN architecture. Works from [50] described a similar approach of using MUX as a function generator, and [51] indirectly inferred weighed average in SC as MAC, but their use cases are not centered on SC MAC operation. Hence, it is necessary to take a step back and look at the absolute basics of SC.

### Theory of MUX in unipolar SC

**Fig. 9.**
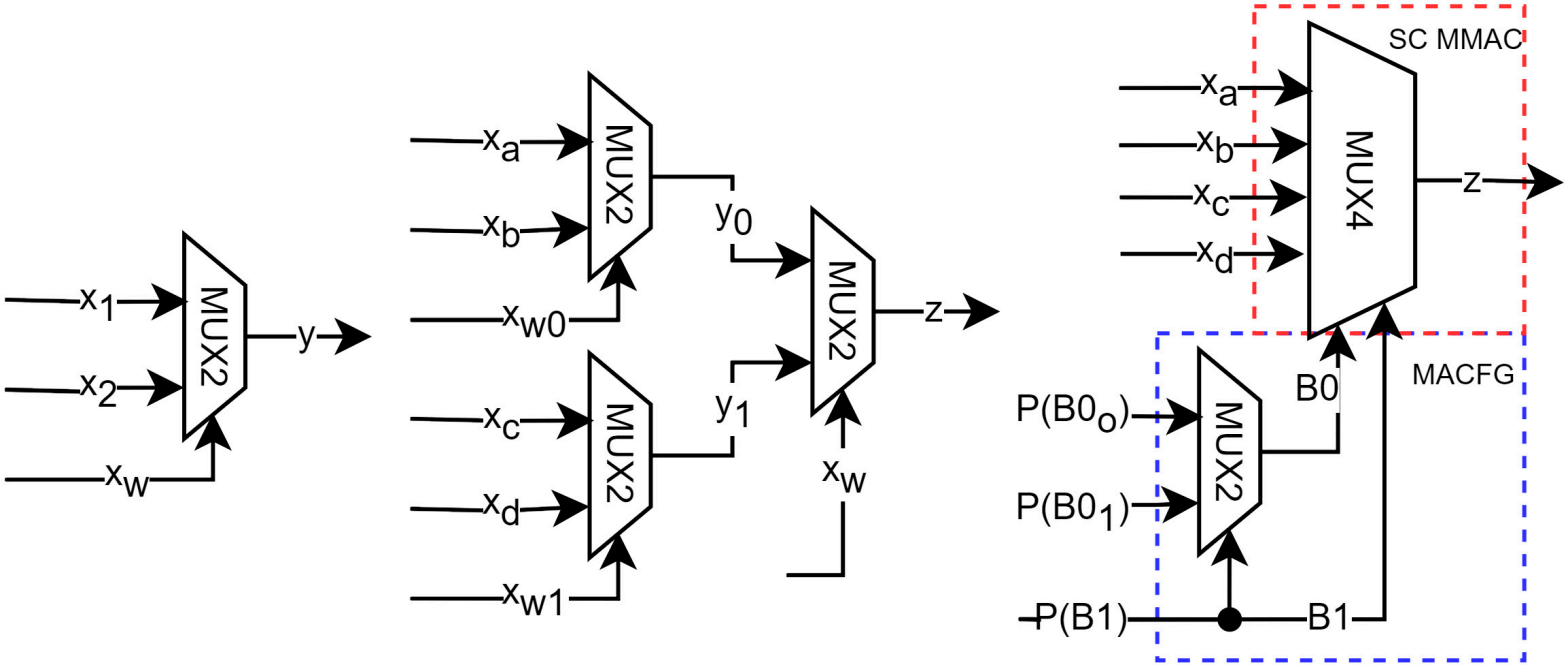
MUX2 (left), MUX4 constitutes 3 MUX2 combinations (middle), and MUX4 MMAC driven by MACFG (right).

Consider the primary form of MUX, i.e., 2-to-1 MUX (annotated as MUX2 / MUXn for *n*-to-1 MUX for the rest of the writing), as illustrated in Fig. 9. SC has 2 operation modes, i.e., unipolar and bipolar. In unipolar mode, the input *x*_1_, *x*_2_, and *x_w_* in probability could be initialized as:

$$\begin{array}{c} x_{1}=P\left(a\right),\;x_{2}=P\left(b\right),\;x_{w}=P\left(w\right),\;{x^{'}}_{w}=1-P\left(w\right) \end{array}$$ (2)

To get the output *y*, simply:

$$y=x_{1}x_{w}^{\prime }+x_{2}x_{w}=P\left(a\right)\left(1-P\left(w\right)\right)+P\left(b\right)P\left(w\right)$$ (3)

When the MUX2 select input is 1, the MUX2 will bypass *x*_2_ stream; otherwise, the *x*_1_ stream will be chosen. If the *x_w_* is 0.5, then:

$$y=\frac{P\left(a\right)+P\left(b\right)}{2}=\frac{x_{1}+x_{2}}{2}$$ (4)

which perfectly functions as a unipolar scaled adder. What if the *x_w_* is 0.2? If so, then:

$$y=0.8P\left(a\right)+0.2P\left(b\right)=0.8x_{1}+0.2x_{2}$$ (5)

which is a simple MAC operation not emphasized in most literature. [2] did mention MUX as a weighted average adder (same as MAC) but limited to MUX2. In fact, the MAC operation could be scaled to MUX4 and beyond. To put into perspective, MUX4 could be made with 3 MUX2s, as shown in Fig. 9. In unipolar mode, the probability equation of MUX4 could be initialized as:

$$x_{a}=P\left(a\right),\;x_{b}=P\left(b\right),\;x_{c}=P\left(c\right),\;x_{d}=P\left(d\right)$$ (6)

$$y_{0}=P\left(a\right)P\left({w^{\prime }}_{0}\right)+P\left(b\right)P\left(w_{0}\right)$$ (7)

$$y_{1}=P\left(c\right)P\left({w^{\prime }}_{1}\right)+P\left(d\right)P\left(w_{1}\right)$$ (8)

$$z=y_{0}P(w')+y_{1}P\left(w\right)$$ (9)

where *y*_0_ and *y*_1_ are the intermediate resultant bitstream functions, and *z* is the final function of the MUX4. If *P*(*w*) =*P*(*w*_0_) = *P*(*w*_1_) = 0.5, then:

$$z=\frac{x_{a}+x_{b}+x_{c}+x_{d}}{4}$$ (10)

Again, it is a 4-to-1 SC scaled adder if the probability is 0.5. Let us assume an arranged truth table in Table 4, where the weight’s sum is 1. Here, the functions of *B*0 are dependent on *B*1. The question is how to program a MUX4 so that the MUX output function matches the truth table’s weights. The solution is straightforward, i.e., swap the *B*0 probability function according to *B*1. Thus, the conditional probability could be derived such that:

$$\begin{array}{c} P\left(B0_{0}\right)=P\left(B0\;\;|\;\;\bar{B1}\right)=\frac{P\left(B0=1,\;B1=0\right)}{P\left(B1=0\right)}=\frac{0.1}{0.4+0.1}=0.2 \end{array}$$ (11)

$$\begin{array}{c} P\left(B0_{1}\right)=P\left(B0\;\;|\;\;B1\right)=\frac{P\left(B0=1,\;B1=1\right)}{P\left(B1=1\right)}=\frac{0.25}{0.25+0.25}=0.5 \end{array}$$ (12)

$$P\left(B1\right)=P\left(B1=1\right)=\frac{0.25+0.25}{0.4+0.1+0.25+0.25}=0.5$$ (13)

where *P*(*B*0_0_) is the first conditional probability of *B*0 when *B*1 is false, and *P*(*B*0_1_) is the second conditional probability of *B*0 when *B*1 is true. **P*(*B*1)* is the overall probability of the most significant bit (MSB) of the MUX4 select input. Then according to the truth table, the output *z* target function could be expressed as:

$$\begin{array}{c} z=P\left(\bar{B0_{0}}\right)P\left(\bar{B1}\right)x_{a}+P\left(B0_{0}\right)P\left(\bar{B1}\right)x_{b}\\ +P\left(\bar{B0_{1}}\right)P\left(B1\right)x_{c}+P\left(B0_{1}\right)P\left(B1\right)x_{d} \end{array}$$ (14)

**Table 4.** Truth table of example MUX4

B1	B0	Multiplier/Weight	MUX4 input
0	0	0.4	*x_a_*
0	1	0.1	*x_b_*
1	0	0.25	*x_c_*
1	1	0.25	*x_d_*

The joint probability function is true only if *B*0 are dependent on *B*1. Alternatively, expanding the previous Eq. 9 also gives:

$$\begin{array}{c} z=P\left({w^{'}}_{0}\right)P(w')P\left(a\right)+P\left(w_{0}\right)P(w')P\left(b\right)\\ +P\left({w^{'}}_{1}\right)P\left(w\right)P\left(c\right)+P\left(w_{1}\right)P\left(w\right)P\left(d\right) \end{array}$$ (15)

By definition of Eq. 6, Eq. 14 is equal to Eq. 15. Thus, it could be inferred that the respective multipliers of Eq. 14 and Eq. 15 are equal, such that:

$$\begin{array}{c} P\left({w^{'}}_{0}\right)P(w')=P\left(\bar{B0_{0}}\right)P\left(\bar{B1}\right),\;P\left({w^{'}}_{1}\right)P\left(w\right)=P\left(\bar{B0_{1}}\right)P\left(B1\right) \end{array}$$ (16)

$$\begin{array}{c} P\left(w_{0}\right)P(w')=P\left(B0_{0}\right)P\left(\bar{B1}\right),\;P\left(w_{1}\right)P\left(w\right)=P\left(B0_{1}\right)P\left(B1\right) \end{array}$$ (17)

To prove that the multipliers are equal, dividing Eq. 16 with Eq. 17 yields:

$$\begin{array}{c} \frac{P\left({w^{'}}_{0}\right)}{P\left(w_{0}\right)}=\frac{P\left(\bar{B0_{0}}\right)}{P\left(B0_{0}\right)},\;\frac{P\left({w^{'}}_{1}\right)}{P\left(w_{1}\right)}=\frac{P\left(\bar{B0_{1}}\right)}{P\left(B0_{1}\right)} \end{array}$$ (18)

Since the numerators could only be the inverted probability function of the denominators in the case of MUX select input, it is proven that *P*(*w*_0_) = *P*(*B*0_0_) and *P*(*w*_1_) = *P*(*B*0_1_). Finally, it also proves that *P*(*w*) = *P*(*B*1). Hence, substituting the calculated probability values into the MUX4 function Eq. 15 yields:

$$z=0.4x_{a}+0.1x_{b}+0.25x_{c}+0.25x_{d}$$ (19)

Thus, it is proven that a MUX4 could perform MAC operation in the SC domain. The SC MMAC operator of MUX4 and beyond has to be driven by an auxiliary function block called the MACFG, as shown in Fig. 9. The MACFG swaps the probability function according to the scheduled weights, matching the selection frequency of the SC MMAC input bitstreams to the target CNN weights in a pseudo-random pattern. Unlike the MUX polynomial function synthesizer from [50] that used tree adders, the MACFG bypassed tree adders, saving FPGA resources.

#### MUX8 as SC MAC operator

Let us take a MUX8 example with an extracted CNN’s 3 × 3 kernel positive weights after the CNN is trained, as shown in Table 5. The weight’s sum shall be 2^8^ − 1 = 255, which is the 8-bit LFSR’s total run length. All the numbers are in fixed points, and dividing by 255 gives the actual floating-point value. From the truth table, the desired output function is:

$$\begin{array}{c} z=\frac{69}{255}x_{13}+\frac{44}{255}x_{23}+\frac{31}{255}x_{12}+\frac{23}{255}x_{33}\\ \;+\frac{17}{255}x_{22}+\frac{8}{255}x_{32}+\frac{63}{255}\left(0\right) \end{array}$$ (20)

**Table 5.** Truth table of example MUX8 select input correspond to the CNN weights and I/Os, and the respective probability functions to be programmed to the MACFG to drive the SC MMAC

Weight	69	44	31	23	17	8	63	0
I/O	*x*_13_	*x*_23_	*x*_12_	*x*_33_	*x*_22_	*x*_32_	0	0
B2	0	0	0	0	1	1	1	1
B1	0	0	1	1	0	0	1	1
B0	0	1	0	1	0	1	0	1
P(B2)	-				88			
P(B1)	-		82		-		183	
P(B0)	-	99	-	109	-	82	-	0

**Fig. 10.**
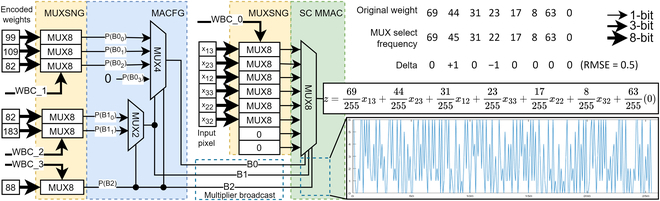
MACFG drives MMAC select input in a pseudo-random pattern that the MUX select frequency matches the CNN weight.

**Fig. 11.**
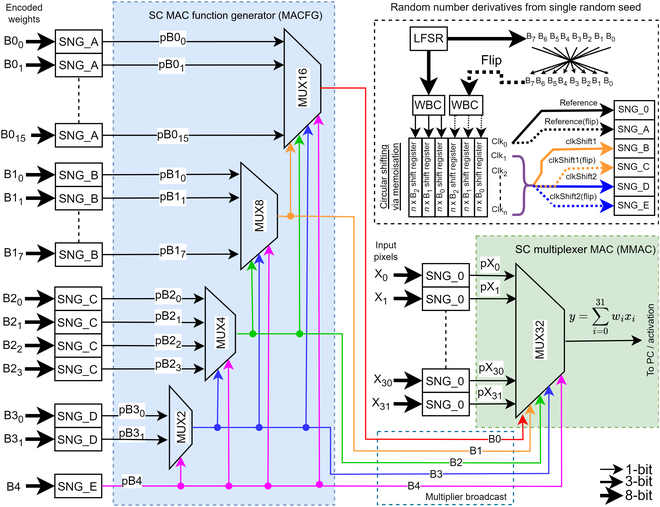
Architecture of a full MUX32 MMAC with MACFG and the LFSR sharing scheme.

However, the weight has to be encoded to program the MACFG to drive the MMAC select input such that the select frequency matches the CNN weights in a pseudo-random pattern. The MACFG output bus has to be extended to 3 bits wide for the select input of the MUX8 SC MMAC such that:

$$P\left(B2\right)=\frac{17+8+63+0=88}{255}=\frac{88}{255}$$ (21)

$$\begin{array}{c} P\left(B1_{0}\right)=P\left(B1\;\;|\;\;\bar{B2}\right)=\frac{31+23}{69+44+31+23}\approx \frac{82}{255} \end{array}$$ (22)

$$\begin{array}{c} P\left(B1_{1}\right)=P\left(B1\;\;|\;\;B2\right)=\frac{63+0}{17+8+63+0}\approx \frac{183}{255} \end{array}$$ (23)

$$\begin{array}{c} P\left(B0_{0}\right)=P\left(B0\;\;|\;\;\bar{B1_{0}}\right)=\frac{44}{69+44}\approx \frac{99}{255} \end{array}$$ (24)

$$\begin{array}{c} P\left(B0_{1}\right)=P\left(B0\;\;|\;\;B1_{0}\right)=\frac{23}{31+23}\approx \frac{109}{255} \end{array}$$ (25)

$$\begin{array}{c} P\left(B0_{2}\right)=P\left(B0\;\;|\;\;\bar{B1_{1}}\right)=\frac{8}{17+8}\approx \frac{82}{255} \end{array}$$ (26)

$$\begin{array}{c} P\left(B0_{3}\right)=P\left(B0\;\;|\;\;B1_{1}\right)=\frac{0}{63+0}=0 \end{array}$$ (27)

where the extended *P*(*B*0_2_) and *P*(*B*0_3_) are the third and fourth conditional probability of *B*0, respectively. Thus, the higher significant bits of the MACFG, as in Fig. 10, recursively feed themselves to swap the lower bitstream’s conditional probability functions, generating a pseudo-random binary selection pattern in the SC MMAC select input. The selection frequency on the SC MMAC select input will eventually converge to the magnitude of CNN weight over the SC run length. Selecting MUX input channels asynchronously multiplies and accumulates the input bitstreams into a single bitstream. The design principle applies to MUX16 and beyond. However, MUX32, as shown in Fig. 11, is the design limit for design automation due to the dimensionality problem in the clocking analysis as described in the next section.

### Optimizing MACFG to minimize error

**Fig. 12.**
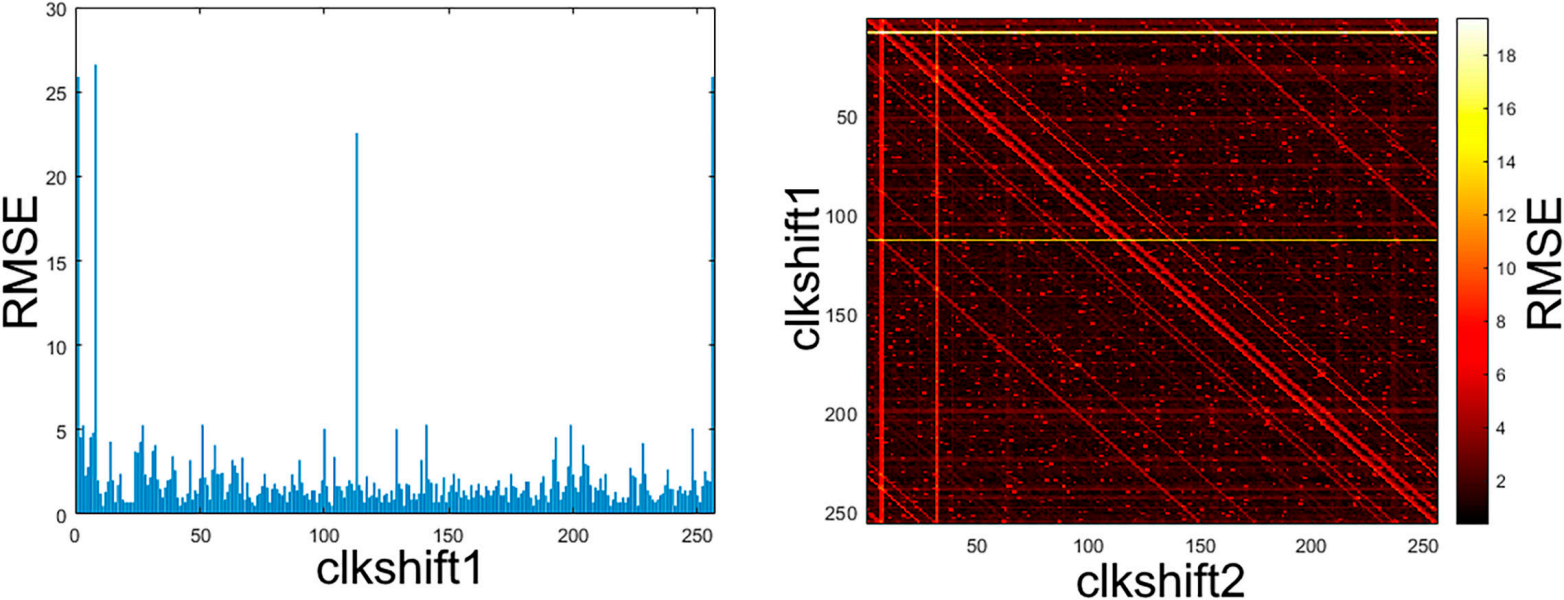
One-dimensional RMSE (MUX4 and MUX8) (left) and 2-dimensional RMSE (MUX16 and MUX32) (right) selection frequency RMSE analysis.

Although SC did compute in the probability domain, the order of the clock source is not purely random. The LFSR random pattern will repeat after a complete state shift cycle of 2*^n^* − 1 clocks. LFSR has received wide research attention in reducing stochastic correlation error in SC. In the research, since 8-bit LFSR has 255 possible states, combining clock shifting and wire permuting [13] contributes to 510 potential LFSR sources. However, there is a need to anchor some LFSR source allocations so that the SC MMAC can be predictably programmed and minimize the stochastic correlation. Prioritizing permuted (i.e., flipped) LFSR allocation is favored to minimize correlation [13]. Each clock-shifted LFSR is paired with its flipped counterpart (in Fig. 11) to reduce the selection frequency analysis complexity. SC MMAC of MUX2 size does not require clock shifting, while MUX4 and MUX8 need one clock-shifting LFSR. MUX16 and MUX32 will have to use a second clock-shifted LFSR, which raises the root mean square error (RMSE) mapping analysis to 2 dimensions. SC MAMC of MUX64 and above are technically possible, but that would raise the RMSE map to 3 dimensions, increasing the simulation time exponentially. Thus, MUX32 is the current design limit for the SC MMAC architecture. Each MACFG is emulated across all possible clocks depending on the target MUX size. The resultant select frequency of the MACFG is aggregated and deducted with the scheduled input-output (I/O) weight for RMSE calculation, as shown in Fig. 10 of example MUX8 MMAC. Especially for 2-dimensional RMSE, as shown in Fig. 12, there are lines of correlation, which suggest that both LFSR of similar clock shifting (and some clock shifts) will have a high correlation, resulting in high RMSE in the SC MMAC select frequencies and must be avoided. Ultimately, the clocks with the lowest RMSE were shortlisted to drive the SC MMAC.

Clock-shifting LFSRs could be achieved using multiple LFSRs of predefined random seeds, but it would use more resources. This research employed the memoization technique via shift registers. Thus, only one LFSR is needed, but it requires one complete SC cycle (255 clocks) warm-up period to iterate all possible clock shifts before the SC circuit can operate. An 8-bit LFSR consumes an 8-bit-wide register. Pairing with the flipped output would cost 16 bits of storage per clock shift. Interestingly, the WBC reduced the data size from 8-bit to 3-bit, pairing with flipped counterpart only costs 6-bit-wide register per clock shift, saving 62.5% of memory needed for LFSR’s circular shifting, as well as reducing 62.5% of wires in broadcasting the signal than the conventional SC. Thus, the WBC improved the scalability of SC in FPGA.

In the case of the Sobol sequence, there are no simple options for random number source sharing like the LFSR, so the MACFG does not support the Sobol sequence. Interestingly, the input pixel is not tightly coupled with the MACFG. Also, the SC MMAC by itself is a MUX, which is not sensitive to stochastic correlation [52], so the Sobol sequence could be fed to the input pixel MUX SNG, i.e., SNG_0 in Fig. 11. Since the LSZ of a binary up counter could be used to generate the Sobol sequence [5], the LSZ could be transcoded to the MSB set for the WBC of the SNG_0 by bit permutation and inversion. The simulation outcomes on the Sobol sequence variant will be further discussed in Results.

### MAC operation with SC MMAC

Bias is sequentially added after the MAC operation in the binary domain. However, the math itself did not describe such restriction. The bias and any multiplier could be out of order in the accumulation stage as long as the final sum is the same. Streamlining bias addition into the SC MMAC is achieved by multiplying with an additional input with a constant high signal while the bias becomes part of the multiplier. Bias addition costs no additional hardware if it fits into the empty slots left in the SC MMAC.

There are some techniques to handle negative signed values in unipolar SC. [19] sorted the weights in ascending order, prioritizing negative counting on the binary counter. Sign-Magnitude SC [29,53] separated sign operation from magnitude arithmetic. Sigmoid activation [39] and shift unary code adder [38] partitioned MAC of opposite signs into 2 separate arithmetic operations, which is being adopted in this research. Sign group splitting in ReLU-activated unipolar SC CNN is possible because:

$$\sum w_{i}x_{i}+b=\sum_{\mathrm{pos}}\;w_{i}x_{i}-\sum_{\mathrm{neg}}\;w_{i}x_{i}+b,x_{i}\geq 0$$ (28)

where *w_i_* is the signed weights in their respective summing group, and *x_i_* is the input image or ReLU-activated features, which could only be in positive values. The input of the subsequent SC element shall be unipolar values only since they also utilize the similar sign group splitting method. The bias will be located in the negative group if it is a negative value.

Multiple SC MMAC channels of different MUX sizes could be grouped into the same PC/APC input. However, there could be extreme cases where the optimization is not ideal because the weight deviation is too massive for a small CNN weight partition. If so, the zero I/O could be filled with a fraction of the unscheduled I/Os weight to fully utilize the MUX input window. The weight splitting does not alter the MAC operation because:

$$\begin{array}{c} w_{i}x_{i}=\left(w_{n1}+w_{n2}+\ldots +w_{\mathrm{nd}}\right)x_{i}\\ \;=w_{n1}x_{i}+w_{n2}x_{i}+\ldots +w_{\mathrm{nd}}x_{i} \end{array}$$ (29)

whereby *w_i_* could be constituted of multiple separable weights. Dynamic weight splitting could improve SC’s mathematical accuracy by allowing weights bigger than the SC run length. A weight of 255 in 8-bit SC is simply an input bitstream bypass. If only one weight in a MUX2 exists, the conventional AND gate is more efficient. Zero I/O is added if no bigger weight is found for weight splitting. In the end, all the MUX input slots could be fully utilized.

#### Advantages of the proposed SC MMAC

Implementing simple logic gates on FPGA LUT is inefficient. The shift unary code adder of [38] based on reduced logic AND–OR gates (or any simple logic gate implementation) has relatively low LUT utilization efficiency on FPGA fabric. The massively paralleled AND gates in the conventional SC also risk wire routing congestion on the FPGA interconnect, potentially failing implementation even if the synthesis process could be successful, rendering the SC on FPGA impractical. Table 6 compares the theoretical advantages of the novel SC MMAC design, targeting Xilinx’s LUT6 architecture.

**Table 6.** Difference of SC MAC design approaches with theoretical FPGA resource usage, targeting Xilinx LUT6

Methods	Input size	LUT count	LUT configuration	LUT function	I/O	Multiplier broadcast bus width
Conventional AND gate (no accumulate)	1	1	(2:1)	AND	1:1	1
	2	1	(4:2)	AND	2:2	2
	4	2	(4:2)	AND	4:4	4
	8	4	(4:2)	AND	8:8	8
	16	8	(4:2)	AND	16:16	16
	32	16	(4:2)	AND	32:32	32
OR-based MAC[38,68]	1	1	(2:1)	AND	1:1	1
	2	1	(4:2)	AND+OR	2:1	2
	4	2	(4:2)	AND+OR	4:1	4
Proposed SC MMAC	1	1	(2:1)	AND	1:1	1
	2	1	(4:2)	AND	2:2	2
			(3:1)	MUX2	2:1	1
	4	1	(5:2) ^a^	MUX2	4:2	1
			(6:1)	MUX4	4:1	2
	8	2	(6:1)	MUX8	8:1	3
	16	4	(6:1)	MUX16	16:1	4
	32	8	(6:1)	MUX32	32:1	5

*^a^* If the multiplier stream is shared

Firstly, the proposed SC MMAC is more resource-efficient than conventional SC architectures. MUX is highly efficient on Xilinx LUT6 FPGA architecture [54]. Suppose the MUX2 multiplier stream is shared, especially in highly paralleled SC MMAC circuits. Then, a LUT6 could fit 2 MUX2s implemented as LUT (5:2) (LUT with 5 inputs and 2 outputs, maximum dual output LUT size allowed on Xilinx FPGA), effectively doubling the LUT6 logic density. A single LUT6 could only fit dual 2-input AND gates, i.e., 2 SC multiplication per LUT6 logic density. In contrast, a LUT6 could fit a MUX4 as an SC MMAC, i.e., 4 SC multiplication and 3 SC addition per LUT6 (>2× logic density of the conventional SC), improving FPGA fabric utilization efficiency.

**Fig. 13.**
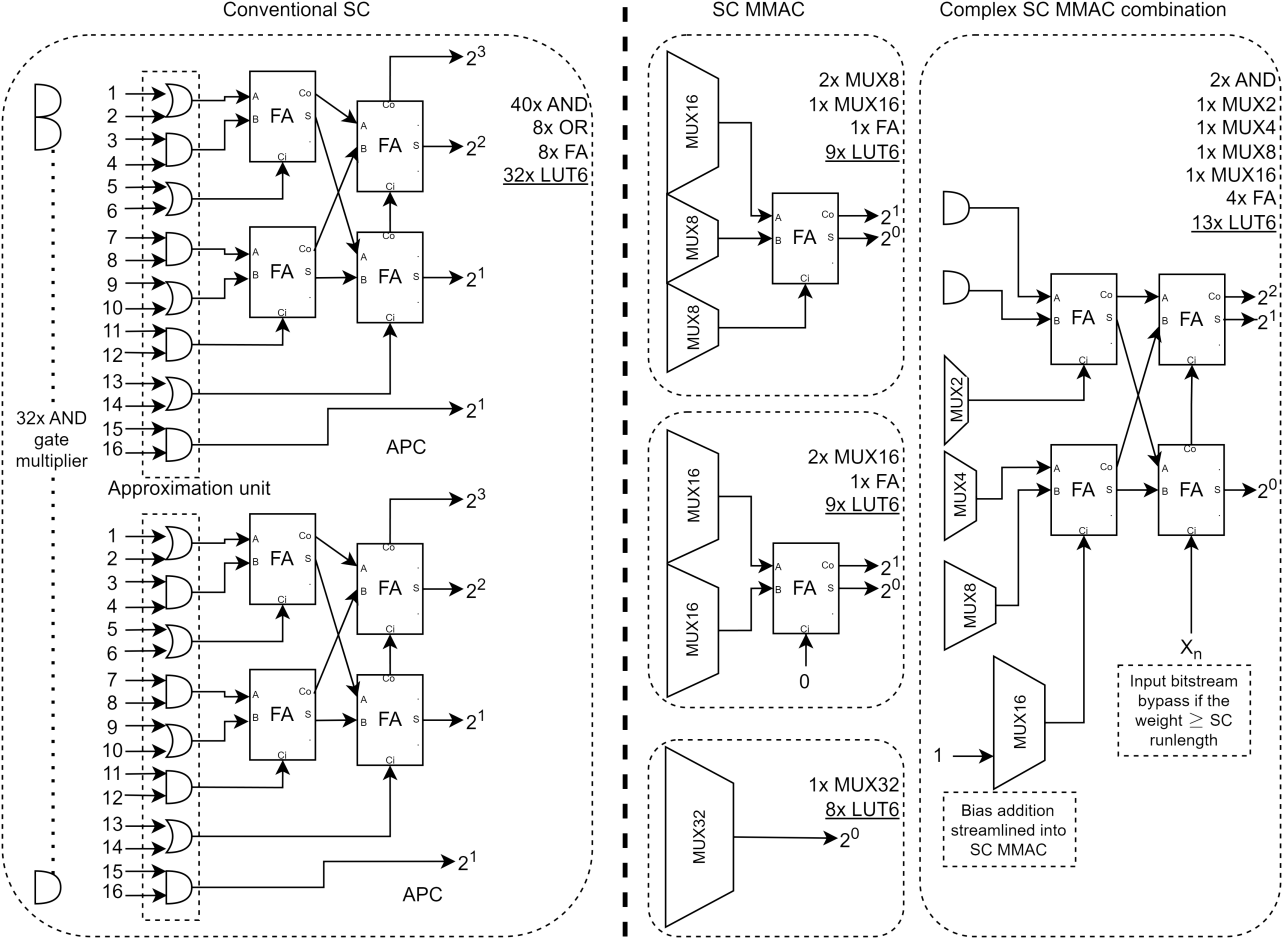
SC MMAC equivalent of 32-input SC MAC hardware (right) compared to the conventional SC approach (left).

Secondly, the SC MMAC could reduce up to 32× stochastic stream as a preaccumulator. It multiplies and partially aggregates the stochastic bitstream before feeding the PC/APC, resulting in 2-staged accumulation. A single MUX16 SC MMAC (costs 4 LUT6s) is functionally equal to 16 AND gates (costs 8 LUT6s) and 16 input APC proposed by [30] (costs another 8 LUT6s, a total of 16 LUT6s) in the conventional SC, reducing the LUT usage by 75%. Most importantly, it solves the inefficiency of large-scale bitstream aggregation in almost all the previous literature. Aggregating 32 bitstreams would need 2 APCs in conventional SC (cost 32 LUTs, including AND gate multipliers). In contrast, the SC MMAC is highly flexible. A highly complex SC MMAC combination still uses >50% less LUT than the conventional SC approach, as illustrated in Fig. 13. The PC/APC is needed only if there is more than one SC MMAC channel. Instead of limiting the summing capacity to 16 inputs, each PC/APC can now expand their summing capacity to a maximum of 32 × 16 = 512 bitstreams with MUX32 as the SC MMAC, improving the SC scalability.

Thirdly, the SC MMAC eradicated SC scaled-adding precision loss problem. The conventional SC MUX2 scaled adder halved the summation (as in Eq. 4), thus requiring double SC run length to preserve the precision information, increasing the overall SC latency to compensate for the precision loss. In contrast, the SC MMAC never scale the summation. Most SC literature separated the arithmetic logic of multiplication and addition. However, the SC MMAC and the MACFG promoted the MUX’s inherent input scaling property (the root cause of the scaled-adding problem) as the SC multiplier while enabling first-stage accumulation at native precision, improving the SC adder accuracy at a much shorter run length. Since the SC MMAC reduced 50% to 97% of the bitstream counts in advance, tiny PCs could perform binary accurate second-stage accumulation, realizing perfectly scaling-free MAC operation. Thus, the SC MMAC avoided resource inefficiency of the integral SC [24], the stochastic correlation problem of the APC [31], and the approximation error of the SAPC [32] altogether.

Meanwhile, [55] mentioned that the correlation-enhanced MUX adder (CeMux) [56] has a weight normalization problem as the number of dot products increases while their range-extended OR-gates also face saturation problems. The SC MMAC, however, does not have any issues above. If the number of smaller dot product increases as the CNN layers deepen, more dot products could fit into a single MUX, favoring the SC MMAC. Expanding multiple SC MMACs and accumulating with FAs (as in Fig. 13) invalidates the above problems altogether. The CeMux also consumed huge MUX sizes by hardwiring multiple MUX channels per input to achieve arbitrary weight scaling. In contrast, the MACFG programmed the weights, consuming only one MUX channel per input on the SC MMAC regardless of the weight’s arbitrary precision.

Lastly, it could MAC 32 parallel stochastic streams with just 5 broadcasted multiplier streams (>6× wire count reduction of the conventional SC), reducing place and route congestion in the FPGA. The separation of the multiplier from the MACFG makes the SC MMAC architecture extremely scalable on FPGA. The MACFG could be shared across all parallel scan windows of the respective CNN filter. For instance, a 6 × 6 × 32 CNN feature layer only requires 32 groups of MACFGs. Each MACFG group could broadcast their output to 6 × 6 = 36 parallel SC MMACs. Thus, the efficiency could only increase as the SC MMAC block gains more parallelism, favoring the CNN algorithm.

### Completing SC CNN with ReLU activation function

The ReLU activation function is favored over TanH due to better CNN model performance, but resolving ReLU as a pure probability function could be challenging. Meanwhile, multiple SC MMACs in parallel result in multiple output bitstreams, but the probability range cannot exceed one on probability. Here, the ReLU activation function could exploit the additional bitstream information to accelerate convergence and improve ReLU function accuracy, much like what had been done by [57] for TanH activation, and this time is for the ReLU function in the SC domain. Before digging into the problem, understanding the SC max pooling component might lead to the solution.

### SC TanH Max as Relu

**Fig. 14.**
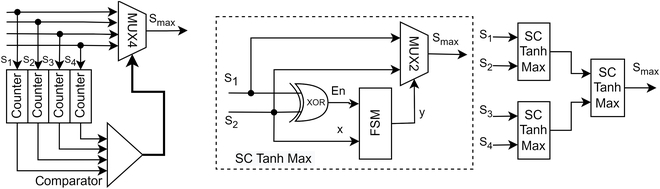
Max pooling with force sampling (left) and XOR gate shutter (right) [48].

CNN max pooling layer selects the biggest value in a scan window to reduce the feature map size while preserving the most important features. It is simple in binary computing to choose the maximum value, but it is not an easy option in SC. Since SC operates in the probabilistic domain, it is unknown which stochastic stream is the biggest in any given clock sample. The only way of making the right decision is to force sampling the streams into binary values. Then, the values will slowly converge to the actual magnitude and then only decide as depicted in Fig. 14, which was the mechanism of [58]. A better design from [33] cleverly used the XOR gate as a sampling shutter for stochastic Tanh FSM. Whenever the XOR gate enables the FSM, the bits from the stream pair could only be either (0,1) or (1,0), i.e., an entangled pair. If it reads 1’s bit on one stream, the other stream must be 0’s bit, and vice versa. The output will eventually converge to select the maximum value by updating the internal state. A newer approach from [36] exploits stochastic correlation to achieve max pooling with a simple OR gate, but the max pooling function is reversed from the standard CNN design flow, i.e., activation should come first, although it might not affect the ReLU activation.

Assume that the SC MMAC only has one stochastic stream for each positive and negative MUX group. In that case, if both streams are fed to the SC Tanh Max function block as in Fig. 14, the function equation changes because now both streams carry the information of positivity and negativity such that:

$$f\left(x\right)=\left\{\begin{array}{cc} +\mathrm{ve}, & P\left(+\right)>P\left(-\right)\\ -\mathrm{ve}, & P\left(+\right)\leq P\left(-\right) \end{array}\right.$$ (30)

where +*ve* and −*ve* denote certain positive and negative values, respectively, while *P*(+) and **P*(−)* denote probability of positivity and negativity, respectively. If the negative stream of the MUX input is forced to zero and makes the default equal magnitude condition zero, the function becomes:

$$\begin{array}{c} f\left(x\right)=\text{ReLU}\left(x\right)=\left\{\begin{array}{cc} +\mathrm{ve}, & P\left(+\right)>P\left(-\right)\\ 0, & P\left(+\right)\leq P\left(-\right) \end{array}\right. \end{array}$$ (31)

**Fig. 15.**
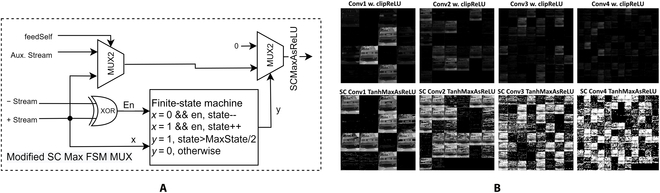
(A) Modified TanH Max as ReLU function. (B) Saturation effect passed down to the subsequent layers if the accuracy problem is ignored. The top feature maps are of the binary domain. The bottom feature maps are of the SC domain with the TanH Max as ReLU architecture.

Hence, an SC Max as ReLU function, as shown in Fig. 15A, is derived, which is slightly modified to accept an auxiliary positive stream aggregated with OR gates for the preliminary design study. If the negative group has more 1’s bit count than the positive group, the output value is deemed negative and shall be clipped to zero. Mathematically speaking, the ReLU decision function in the SC domain could be written as:

$$\begin{array}{c} \text{ReLU}\left(x\right)=\left\{\begin{array}{cc} \;+\mathrm{ve}, & \sum P\left(+\right)>\sum P\left(-\right)\\ \;0, & \sum P\left(+\right)\leq \sum P\left(-\right) \end{array}\right. \end{array}$$ (32)

However, decision-making is just part of the ReLU operation. It has to return the accurate positive amplitude in the end. Multiple stochastic streams could be added with OR gates if the magnitude is very small [2]. In probability, adding very small stochastic numbers could be approximated as:

$$\begin{array}{c} P\left(a\right)+P\left(b\right)=P\left(a\cup b\right)-P\left(a\cap b\right)\\ \approx P\left(a\right)\;OR\;P\left(b\right),\;\lim_{\left(a,b)\to (0,0\right)}P\left(a\cap b\right)=0\; \end{array}$$ (33)

However, as the number of parallel bitstreams increases, the chance the bits intersect increases. If the summation accuracy is ignored, simply accumulating multiple bitstreams with OR gates will eventually saturate the output [59]. The saturation effect is already visible in the SC CNN first layer output, as shown in Fig. 15B of the simulated feature maps. Since the resultant bitstream is passed down to the next SC CNN layer, the saturation effect accumulates, and the outcomes only worsen as the CNN layer goes deeper.

### Novel SC ReLU architecture

It is observed that a state register could store the required amplitude information. The state could be subtracted if the delta of the summation is negative, otherwise is added. A 1’s bit has to be pushed to the stochastic output stream as a ReLU activation function. If the state is bigger than the state’s middle threshold, then the state shall be deducted by 1. The vectored mathematical expression could be described as follows:

$$\left[y,\;s\right]=\left\{\begin{array}{l} \left[1,\;s-1\right],\;s+\left(\sum P\left(+\right)-\sum P\left(-\right)\right)>2^{n-1}-1\\ \left[0,\;s\right],\;s+\left(\sum P\left(+\right)-\sum P\left(-\right)\right)\leq 2^{n-1}-1 \end{array}\right.$$ (34)

where *n* is the binary resolution of the state *s* and *y* is the ReLU output. The SC BReLU will compensate for the output and update the internal state as soon as the state starts biasing. It also breaks the output stochastic stream correlation relationship to improve the quality of randomness of the next SC block. It eliminates the need for the probability estimator [26,60] since it is not required to know the exact magnitude of the bitstream. Moreover, the decision convergence time is much faster than SC Max As ReLU since it accepts multiple bitstreams information. However, the state in Eq. 34 has to update twice if the inequality operation is true, consuming 2 clocks and bottlenecking the entire SC circuit. Instead, the state update could be pipelined to the next clock cycle by using a residue bit *r* such that:

$$r_{t+1}=\left\{\begin{array}{cc} 1, & s_{t}+\left(\sum P\left(+\right)-\sum P\left(-\right)\right)-r_{t}>2^{n-1}-1\\ 0, & s_{t}+\left(\sum P\left(+\right)-\sum P\left(-\right)\right)-r_{t}\leq 2^{n-1}-1 \end{array}\right.$$ (35)

where *t* is the SC clock cycle. Interestingly, the MSB of the state *s* exactly equals the logic of 2^*n* − 1^ − 1. Assuming the state resolution *n* = 5, whenever the sum is 16 or above (b’10000 in binary radix), it means that the MSB of the state is set. So, the residue bit could be extracted from the state’s MSB. Also, the residue bit is the required output of the SC BReLU, logically speaking. Thus, the overall mathematical expression could be reduced to:

$$s_{t+1}=s_{t}+\left(\sum P\left(+\right)-\sum P\left(-\right)\right)-r_{t}$$ (36)

$$\text{BReLU}\left(x\right)=\mathrm{MSB}\left(s_{t+1}\right)=r_{t+1}$$ (37)

Ultimately, the SC BReLU output is just the MSB of the resultant state. Only one clock cycle is needed to compute the ReLU function in the SC domain.

**Fig. 16.**
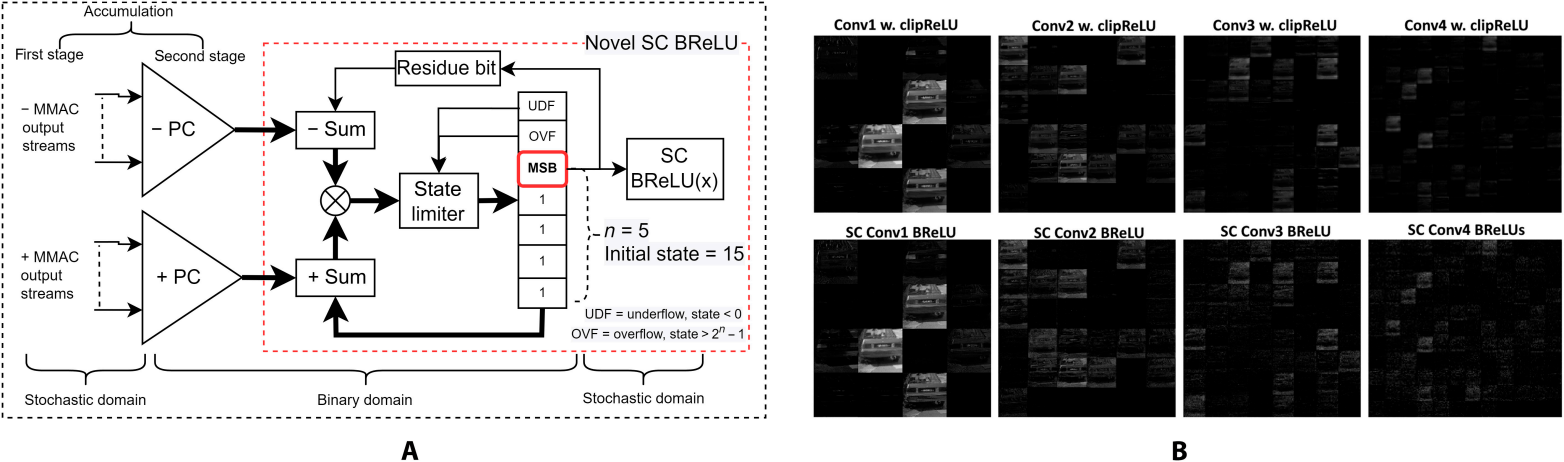
(A) Novel devised SC BReLU. (B) Binary CNN computed convolution feature map (top) versus SC CNN convolution feature map (bottom). The novel SC BReLU successfully executed the ReLU function, resembling binary domain output.

Solving the nonlinear ReLU function in a probabilistic manner is challenging. Much recent literature retrieves the actual magnitude before the ReLU, inducing huge computational latency. The novel SC BReLU, however, allow bitstream transfer to the next SCCNN layer immediately because it only measures the biases of random distribution. A bigger state size provides more room for biasing, leading to better convergence performance. The state limiter keeps the state’s magnitude at a minimum of zero (in the case of underflow) or a maximum of 2*^n^* − 1 (in the case of overflow). SC ReLU function took a free ride with the state’s MSB set, which is similar to [33], but the SC BReLU’s binary adder superseded the FSM’s decision mechanism by casting the decision function as an arithmetic operation via the residue bit feedback loop. In fact, the binary adder itself is the SC ReLU activation function, achieving truly zero hardware for SC ReLU activation since the PC’s outputs have to be added anyway. The state’s MSB is the natural product of the SC ReLU activation function, as shown in Fig. 16A of the devised SC BReLU block. As shown in Fig. 16B, the initial simulation results on the convoluted feature maps between the 2 computing domains are virtually identical, with no sign of saturation as in the previous Fig. 15B. The outcome signifies that the novel SC MMAC operator with the novel devised SC BReLU could replicate the binary clipped-ReLU function in the SC domain.

**Fig. 17.**
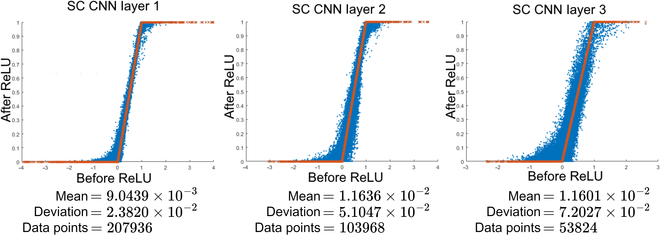
Interlayer feedforward error distribution. Binary clip ReLU (orange crosses) versus SC CNN BReLU (blue dots).

If the previous work of SC ReLU architecture from [35] is compared, they claimed that their SC ReLU had a mean error of 0.031 for a 1,024-clock SC run length. However, Fig. 17 shows that the clipped ReLU response of the SC CNN Layer 1 of the proposed SC BReLU architecture achieved a mean error of 0.009, i.e., 3.4× accuracy improvements at a much shorter 256-clock SC run length. Even if SC gets more noise in the subsequent layer, it is still more accurate than [35]. One reason is that [35] was on bipolar SC, which naturally penalizes the SC accuracy [61]. The more profound difference is that the novel SC BReLU took multiple bitstream information to achieve a better ReLU response. SC noise might not be the critical demerit, especially for the CNN classification task, whereby only the class of maximum amplitude matters.

The initial simulation outcomes from Fig. 17 show that it has virtually zero latency when the stochastic streams from the former layer are passed down to the next layer. The implication is huge. Full implementation of *n*-bit SC CNN inferencing feedforwards through all layers only took 2*^n^* − 1 clock cycles regardless of the CNN size and depth unless the bitstream is forcibly sampled. The stochastic bit is as if it is an integral part of the computing and needs no memory to store the intermediate result, i.e., in-memory computing. In reality, some memory is required for pipelining implementation, inducing some latency. Still, the fact that 8-bit SC completed all CNN layers in around 255 clock cycles is of huge interest.

### SC-oriented CNN design

**Fig. 18.**
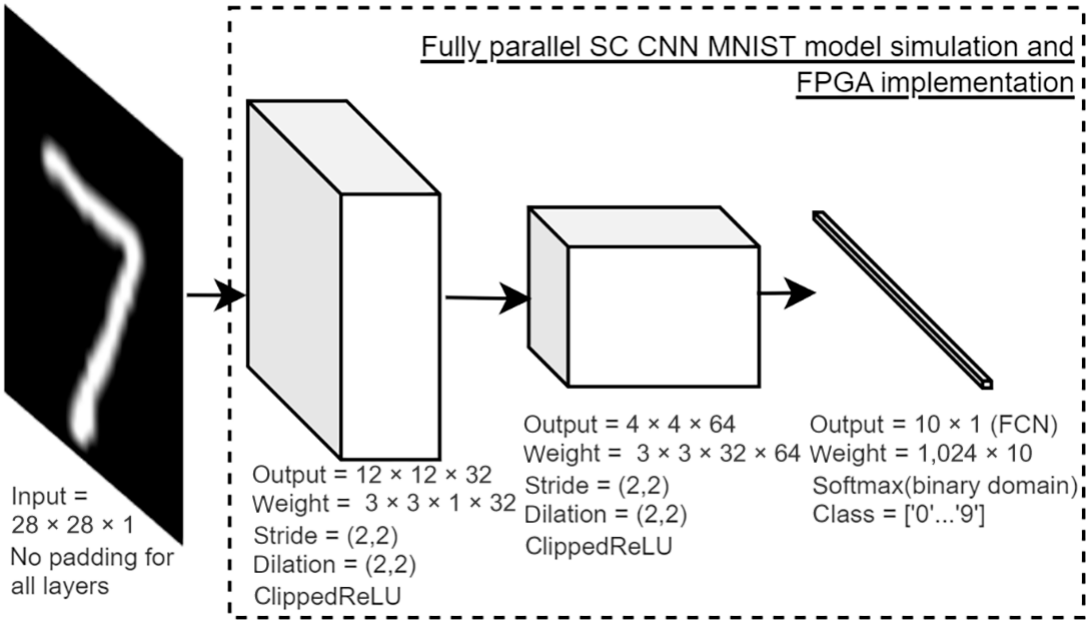
Simple SC-efficient CNN model for handwriting MNIST classification simulation and FPGA implementation.

Although the pooling layer helps improve feature spatial invariance [62], abandoning 75% of the bitstream information in a typical 2 × 2 max pooling layer might not help the SC BReLU bitstream convergence while consuming SC resources. Also, SC is already memory efficient, making the pooling layer in the SC domain less functional. Worse still, SC max pooling suffers approximation errors [36] while MUX scaled-adder in the SC average pooling induces precision loss. Thus, the pooling operation is superseded with convolution strides and dilation operations, effectively downsizing the feature while having a bigger effective field of view of the scan windows, improving feature capture. Even better, stride and dilation cost no additional SC hardware because they are just bitstream wire rerouting. A simple CNN model as in Fig. 18 is FPGA SC-oriented design. The stride and dilation operation limited the CNN model to 2 convolution layers, and the number of filters is the optimum design with good accuracy before the SC architecture is proven feasible on FPGA, achieving a respectable 98.36% handwriting MNIST classification accuracy with adaptive momentum optimization algorithm (Adam) [63] in the binary domain. Handwriting MNIST dataset consists of handwritten images for a single digit from 0 to 9, having 60,000 training sets and 10,000 test sets.

The last FCN layer is of 1-dimensional convolution, flattening the 4 × 4 × 64 pixel features from the second CNN layer into 1,024 input plus bias. The output of MACFG, in this case, is broadcasted to one SC MMAC only, making FCN implementation less resource efficient. Still, the SC MMAC is beneficial as a preaccumulator in reducing the overall output bitstream count in Layer 3, i.e., 1,024 inputs down to just 80+ output bitstreams on each Layer 3 MUX sign group before feeding the PC, greatly reducing the required PC size. Otherwise, parallel counting thousands of bitstreams would be costly.

### Achieving high-performance SC CNN inferencing on FPGA

Deeply cascading the entire SC CNN functions is impractical if the operating clock period is less than the signal path delay. Although SC is a different computing domain, it still benefits from traditional logic elements such as flip-flops (FF) for pipelining. The pipelining implementation causes the global signal to be out-of-phase with local SC CNN intellectual property (IP) as more IP blocks are cascaded. Thus, the IP has to implement a signal relay function so that the global signal can be in-phase with the respective IP’s output. Relaying signal on Xilinx FPGA is highly efficient with its primitive shift register LUT (SRL), whereby the LUT itself could be implemented as a 32-bit shift register to reduce route congestion. Resetting or writing memory must need a dedicated clock. One complete 8-bit SC cycle driven by LFSR only has 255 clocks. However, there is no timing window for the SC CNN to reset its internal registers for feedforwarding a new image for the next SC cycle, especially the SC BReLU function block. Otherwise, the SC CNN FPGA hardware cannot be reused to compute a new batch of data since the registers were biased to the former data batch. An additional clock cycle is explicitly injected to reset the necessary registers, resulting in 256 clocks per SC cycle. The pipelining latency could be overlooked in large image batch feedforward, achieving true 256-clock complete 8-bit SC execution.

#### Eliminating interface bottlenecking and enabling EDT

The data transfer must be completed within the SC’s 256 timing window so that the SC CNN can continuously process a new batch of data. The SC CNN for the handwriting MNIST classification took 13 × 13 × 1 flattened pixels (accounting for strides and dilation operations), or 169 bytes of data could be transferred via a 64-bit bus in under 22 clocks. The output data stream only has ten 16-bit integer class outputs, taking 10 clocks to complete data transfer with a 16-bit data bus. Thus, the I/O consumed at least 80 FPGA I/O pins and additional ports to interact with external systems.

A 22-clock complete data transmission allows SC’s inherent EDT capability of a minimum of 23 clocks (one clock for reset signal), terminating the SC execution cycle earlier once the output converges to the desired outcomes, pushing speed and energy efficiency further. The LFSR source is modified to accept an 8-bit user input value to enable EDT. It holds a copy of 255 clock cycle WBC register values after an initial 255 clock cycle warm-up period. When the EDT is enabled, a counter will track the SC cycle. It rewrites the active register with the copy register when the counter matches the user-defined EDT value, resetting the WBC register to the initial LFSR timing to restart the SC cycle.

#### Compressing CNN’s weights in the SC domain

The MACFG map has already compressed some of the CNN weights. Take the scheduled weight map in Table 7, for instance. The scheduled weight is in descending order because they are sorted before the scheduling. The software picked the right one and grouped them in a perfect 255-clock window. The MUX32 has 5 levels of MACFG encoded weight, i.e.,* B*0, *B*1, *B*2, *B*3, and *B*4, each containing the probability function of the MACFG. To decode the CNN weight for SC MMAC operation, e.g., the last weight of 1, multiply the probability function gives:

$$\begin{array}{c} w_{31}=B0_{15}B1_{7}B2_{3}B3_{1}B4=\frac{128}{255}\\ \times \frac{73}{255}\times \frac{66}{255}\times \frac{86}{255}\times \frac{80}{255}\approx \frac{1}{255} \end{array}$$ (38)

**Table 7.** SC CNN layer 3 Filter 1 -MUX group encoded MACFG weights

Scheduled CNN weights on example MMAC of MUX32
Weight 1-8	16	15	14	13	12	12	11	11
	Weight 9-16	10	9	9	9	9	9	8	8
Weight 17-24		8	7	7	7	6	6	6	6
	Weight 25-32	5	5	5	5	3	2	1	1
Clock source		MACFG level	Encoded weights							
Flip LFSR	B0 (1-8)	123	123	128	128	121	128	128	119
	B0 (9-16)	128	128	128	128	128	128	102	128
ClkShift1	B1	119	122	124	120	123	128	128	73
Flip ClkShift1	B2	113	122	115	66				
ClkShift2	B3	103	86						
Flip ClkShift2	B4	80							

**Fig. 19.**
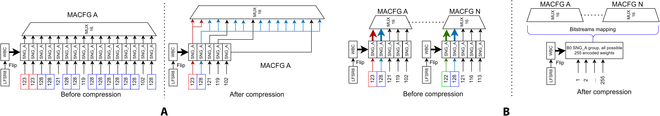
(A) MACFG encoded weight L1 compression scheme. (B) L2 compression scheme.

Notably, there are a lot of repeated weights. Since they use the same LFSR clock source for the individual level, they could be compressed into a single weight to be shared among them, reducing the number of SNGs, namely the L1 compression scheme. The compression ratio depends on the scheduled weights arrangement. Bigger MUX could have a better compression ratio, and smaller MUX has relatively more uniquely encoded weights and is harder to reduce further. The L1 compressed encoded weight will have the MACFG configuration, as shown in Fig. 19A.

If the clock source of the MACFG Level B0 encoded weight is observed, all of the weights across all SC CNN shared the same LFSR clock source, yet the encoded weights could be duplicated across different MACFGs. Instead of repeating the similar encoded weight across different MACFGs, they could be aggregated into a single B0 SNG group that solely supplies the stochastic streams to all MACFGs across all layers and filters, namely the L2 compression scheme as illustrated in Fig. 19B. The L2 compression scheme effectively compressed at least 50% of the encoded weights of the entire SC CNN, as shown in Table 8. The B0 encoded weight could then be mapped to the B0 SNG group block to generate the stochastic streams of all possible encoded weights. MACFGs Level B1 onward cannot perform L2 compression because the clock sources differ. Note that the lower the compression ratio, the more it compresses relative to its original size. L2 compression is favored because it is easier to implement.

**Table 8.** MACFG weight compression level analysis on a sample SC CNN. The actual LUT usage is 75% less than expected because CNN’s weights could be fixed as Boolean logic with 3-bit WBC.

MACFG’s MUX SNG	Original	After scheduling	L1 compression	L2 compression
SC CNN Layer 1	432	400 (92.59%)	397 (91.90%)	157 (36.34%)
SC CNN Layer 2	4,608	4,282 (92.97%)	3,897 (84.57%)	1,969 (42.73%)
SC CNN Layer 3	18,432	17,402 (94.41%)	13,919 (75.52%)	8,243 (44.72%)
SC CNN Layer 4	73,728	69,353 (94.07%)	47,338 (64.21%)	33,465 (45.39%)
Shared B0	-	-	-	255
Components	ASIC’s WBG	MUX SNG	WBC (fixed CNN weights)	
LUT count	3	2(−33%)	0.5(−83%)(−75% MUX SNG)	

Each MUX SNG is expected to utilize 2 LUT6s as MUX8, i.e., 510 LUTs, for the Shared B0 block. However, the weights are fixed and could be hardwired as Boolean logic, bypassing the MUX SNG implementation and consuming only 127 LUTs (75% less) as in Table 9. Since the MACFG B0 weights could be hardwired, it could be inferred that the actual resource requirements for the rest of MACFG’s weights are 75% less than estimated in addition to the L2 compression. The WBC is highly advantageous here because the input is only 3-bit wide. Thus, the LUT6 could be configured for dual output LUT (3:2), doubling the FPGA’s logic density. Otherwise, each MUX SNG would have consumed 2 LUTs for every SC CNN’s weight, not to mention the 3 LUTs of an ASIC’s WBG [22]. Simply put, the WBC also reduced the resource required by 83% than the brute force transcoded ASIC’s WBG, greatly improving the resource efficiency in implementing fixed SC CNN weights on FPGA.

**Table 9.** Resource utilization of fully parallel handwriting MNIST SC CNN on Kintex7 XC7K325T FPGA

SC CNN Layer 1 (latency = 2)	Conv output map size	LUT	SRL	FF	CP (ns)
Filter 1-16	6 × 6 × 16	10,032	0	9,020	2.203
Filter 17-32	6 × 6 × 16	10,379	0	5,534	2.308
	Total/max	20,411		14,554	2.308
SC CNN Layer 2 (latency = 3)	Conv output map size	LUT	SRL	FF	CP (ns)
Filter 1-16	4 × 4 × 16	30,955	896	11,369	3.275
Filter 17-32	4 × 4 × 16	30,324	0	6,172	3.298
Filter 33-48	4 × 4 × 16	30,459	0	6,159	3.299
Filter 49-64	4 × 4 × 16	30,243	0	6,144	3.235
	Total/max	122,877		29,844	3.299
SC FCN Layer (latency = 3)	Conv output map size	LUT	SRL	FF	CP (ns)
Filter 1-5	1 × 5	4,580	862	6,110	3.989
Filter 6-10	1 × 5	3,768	0	2,175	4.426
	Total/max	9,210		8,285	4.426
Components	LUT	FF	LUT (%)	FF (%)	CLB MUX
LFSR	3,682	4,136	1.81	1.01	F7MUX
SNGS0	127	0	0.06	0	36.75%
MUXSNG	401	4,508	0.2	1.11	
I/O Remap	0	0	0	0	F8MUX
SC CNN L1	20,411	14,554	10.02	3.57	21.59%
SC CNN L2	122,877	29,844	60.29	7.32	
FCN	9,210	8,285	4.52	2.03	F9MUX
16-bit Accumulator	294	536	0.14	0.13	NA
Total (synthesis)	157,002	61,863	77.04	15.18	
Total (implement)	153,151	55,395	75.15	13.59	

CP, critical path; CLB, configurable logic block; NA, not applicable.

#### SC CNN IP resource estimation, integration, and implementation

Some assumptions are applied to estimate the resource usage. In the traditional AND gate approach, all CNN weights use B0 SNG to generate multiplier bitstreams. 16-input APCs and the required tree adders are accounted to sum up the stochastic bit count before feeding the SC BReLU block. To estimate the resources of the proposed SC MMAC and MACFG, the number of MUXs is computed and aggregated. The MACFG output could be broadcasted, so only one MACFG group is needed for each layer. One part of the components cover single-pixel output for the entire CNN channel, and multiplying the LUT6/part with the output width and height reveals the total resource estimation for a single CNN layer, as shown in Table 10. It is estimated that the proposed SC MMAC architecture could save up to 2.967× of resources compared to the conventional AND gate method. The resources used to aggregate the SC bitstream are enormous in the conventional method. In contrast, the SC MMAC greatly reduced resources with the 2-staged bitstream aggregation technique while achieving binary accurate bit counting. The MACFG also reduced the actual multiplier required, reducing the overall resource usage.

**Table 10.** Resource utilization estimation before and after optimization

On conventional AND gate					Proposed SC MMAC MACFG					
Components		LUT6 /part	Part count	Sum	Components			LUT6 /part	Part count	Sum
Input	MUX SNG	2	13 × 13	338	Input	MUX SNG		2	13 × 13	338
L1	AND gate	(3 × 3 + 1) × 32/2	6 × 6	5,760	L1	MMAC		151	6 × 6	5,436
	Weight	0	0	0		MACFG	WBC	0.5	76	38
	APC	1 × 8 × 32	6 × 6	9,216			MUX	77	1	77
	5-bit tree adder	0	6 × 6	0		PC		72	6 × 6	2,592
	BReLU	14 × 32	6 × 6	16,128		BReLU		14 × 32	6 × 6	16,128
L2	AND gate	(3 × 3 × 32+1) × 64/2	4 × 4	147,968	L2	MMAC		4,639	4 × 4	74,224
	Weight	0	0	0		MACFG	WBC	0.5	7,594	3,797
	APC	19 × 8 × 64	4 × 4	155,648			MUX	4,636	1	4,636
	5-bit tree adder	18 × 4 × 64	4 × 4	73,728		PC		1,036	4 × 4	16,576
	BReLU	14 × 64	4 × 4	14,336		BReLU		14 × 64	4 × 4	14,336
**L3**	AND gate	(4 × 4 × 64 + 1) × 10/2	1	5,125	**L3**	MMAC		2,547	1	2,547
	weight	0	0	0		MACFG	WBC	0.5	3,774	1,887
	APC	65 × 8 × 10	1	5,200			MUX	2,546	1	2,546
	5-bit tree adder	64 × 4 × 10	1	2,560		PC		1,512	1	1,512
	16-bit Adder	15 × 2	10	300		16-bit Adder		15 × 2	10	300
Global	B0 WBC	0.5	256	128	Global	B0 WBC		0.5	256	128
Total LUT6 with conventional AND gate (estimated)	436,435	Total LUT6 with proposed SC MMAC (estimated)					147,098

Resource utilization data from Table 9 shows that the actual resource usage is 4.1% more than estimated. Although the SC CNN Layer 1 only has [3 × 3 × 1(input kernel) × 6 × 6 × 32(output feature) × 2(MAC) + 6 × 6 × 32(output feature) × 2(bias + ReLU)] / 20411 LUT = 1.1288 OP/LUT logic density (including PC and BReLU) due to low initial input dimension, the SC CNN Layer 2 and the FCN layer achieved 4.8168 OP/LUT (including PC and BReLU) and 2.2248 OP/LUT (including PC), respectively, exceeding the conventional SC AND gate array’s theoretical efficiency limit of 2 OP/LUT (excluding PC and BReLU). Such hardware utilization efficiency are untouchable in the binary computing domain. APC is avoided because it breaks the correlation relationship with MATLAB simulation while inducing summation errors. Besides, Xilinx configurable logic block (CLB) architecture efficiently implements FA with hardwired carry chains, simplifying the synthesis workflow. The implementation shows that the SC MMAC could fit both CNN and FCN layers as long as MAC operation in the SC domain is concerned. Meanwhile, the actual hardware MUX in the CLB, i.e., F7MUX and F8MUX, had widely been utilized in the SC CNN hardware due to the large-scale MUX implementation by the SC MMAC and MACFG. MUX8 and above would use the F7/F8MUX, maximizing the FPGA fabric resource utilization, which would otherwise remain unused in the conventional SC logic. Newer Xilinx Ultrascale FPGA has F9MUX to fit MUX32 in a single CLB, potentially making the SC MMAC architecture even more scalable.

**Fig. 20.**
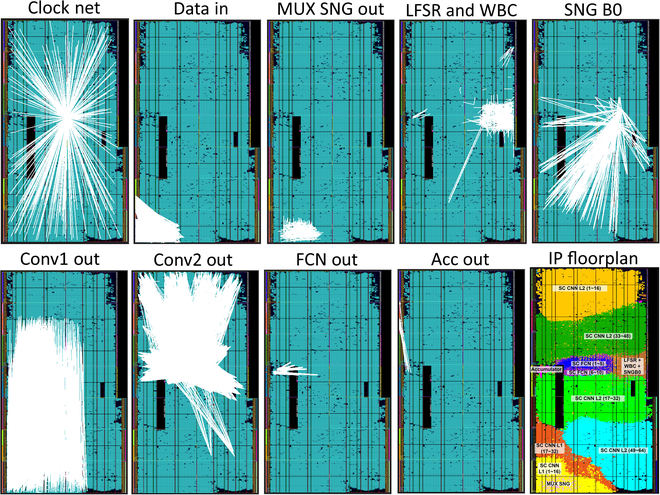
Implemented SC CNN’s signal routes and FPGA floorplanning.

**Fig. 21.**
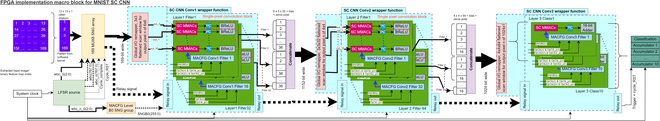
SC CNN FPGA architecture for handwriting MNIST classification.

Each SC CNN layer is partially wrapped as a C++ function wrapper to achieve partial implementation via Xilinx Vitis High-Level Synthesis (HLS) while exploiting modern multicore processors to speed up IP synthesis with multiple HLS instances before integrating the IPs as a fully parallel SC CNN on FPGA. Simply concatenating the output bitstreams in the IP integration stage would join the partial IPs. The SC CNN hardware is practical only if the implementation is successful, i.e., no timing violations (worst negative slack and worst hold slack are non-negative) and all logic and signals are placed and routed on the FPGA fabric, as shown in Fig. 20 of the visualized signal routes and placed IPs. The architecture of the fully parallel SC CNN and the mechanism of cascading SC CNN IPs are shown in Fig. 21. 

## Data Availability

Data is available (with condition) upon request.
